# Total synthesis of natural products using hypervalent iodine reagents

**DOI:** 10.3389/fchem.2014.00115

**Published:** 2015-01-05

**Authors:** Gaëtan Maertens, Chloé L'Homme, Sylvain Canesi

**Affiliations:** Laboratoire de Méthodologie et Synthèse de Produits Naturels, Département de Chimie, Université du Québec à MontréalMontréal, QC, Canada

**Keywords:** total synthesis, hypervalent iodine, alkaloids, dienone, natural products

## Abstract

We present a review of natural product syntheses accomplished in our laboratory during the last 5 years. Each synthetic route features a phenol dearomatization promoted by an environmentally benign hypervalent iodine reagent. The dearomatizations demonstrate the “aromatic ring umpolung” concept, and involve stereoselective remodeling of the inert unsaturations of a phenol into a highly functionalized key intermediate that may contain a quaternary carbon center and a prochiral dienone system. Several new oxidative strategies were employed, including transpositions (1,3-alkyl shift and Prins-pinacol), a polycyclization, an *ipso* rearrangement, and direct nucleophilic additions at the phenol *para* position. Several alkaloids, heterocyclic compounds, and a polycyclic core have been achieved, including sceletenone (a serotonin reuptake inhibitor), acetylaspidoalbidine (an antitumor agent), fortucine (antiviral and antitumor), erysotramidine (curare-like effect), platensimycin (an antibiotic), and the main core of a kaurane diterpene (immunosuppressive agent and stimulator of apoptosis). These concise and in some cases enantioselective syntheses effectively demonstrate the importance of hypervalent iodine reagents in the total synthesis of bioactive natural products.

## Introduction

Natural product synthesis is a subject of fascination for many chemists because of the inherent elegance of the synthetic procedure and because many of these compounds have demonstrated important bioactivities, including the noteworthy examples taxol and paclitaxel (Goodman and Walsh, 2001). Indeed, several of the natural products generated by Nature result from a long evolution of plants and microorganisms to develop defenses against pathogens and hostile environments. This long evolution is probably the key to the diverse bioactivity displayed by these compounds and the reason why many pharmaceutical compounds are isolated, inspired, or derived from natural sources. However, the total synthesis of natural products remains a challenge despite the powerful methodologies developed during the last century, and efficient new tools are still a necessity, not only for optimization of the synthetic process in terms of number of steps, yield, atom economy, and reduced environmental burden but in many cases to merely produce the desired architecture at all. Total synthesis has been the focus of many groups, enabling the advancement of basic knowledge as well as the development of new drugs and the creation of efficient tools and strategies to produce complex natural architectures. In this present article, we describe several of our own natural product syntheses accomplished during the past 5 years. The methods are based on phenol dearomatization mediated by hypervalent iodine reagents, and the target molecules are divided into two classes. The first class consists of molecules containing at least one quaternary carbon center obtained using methodologies developed in our laboratory and based on the phenol dearomatization process. Successful formation of a quaternary carbon involves a transposition (1,3-alkyl shift or Prins-pinacol), a polycyclization, or an *ipso* rearrangement. The second class represents molecules containing a trisubstituted alkene moiety obtained indirectly through hetero-nucleophilic addition followed by elimination. The strategies presented here have enabled us to synthesize alkaloids such as sceletenone (a serotonin reuptake inhibitor), acetylaspidoalbidine (an antitumor agent), fortucine (antiviral and antitumor), erysotramidine (curare-like effect), the heterocyclic antibiotic platensimycin, and the main tetracyclic core of a kaurane diterpene. Depending on the strategy employed, the molecules obtained may be racemic or enantiopure.

## The aromatic ring umpolung concept

**Figure 1:**
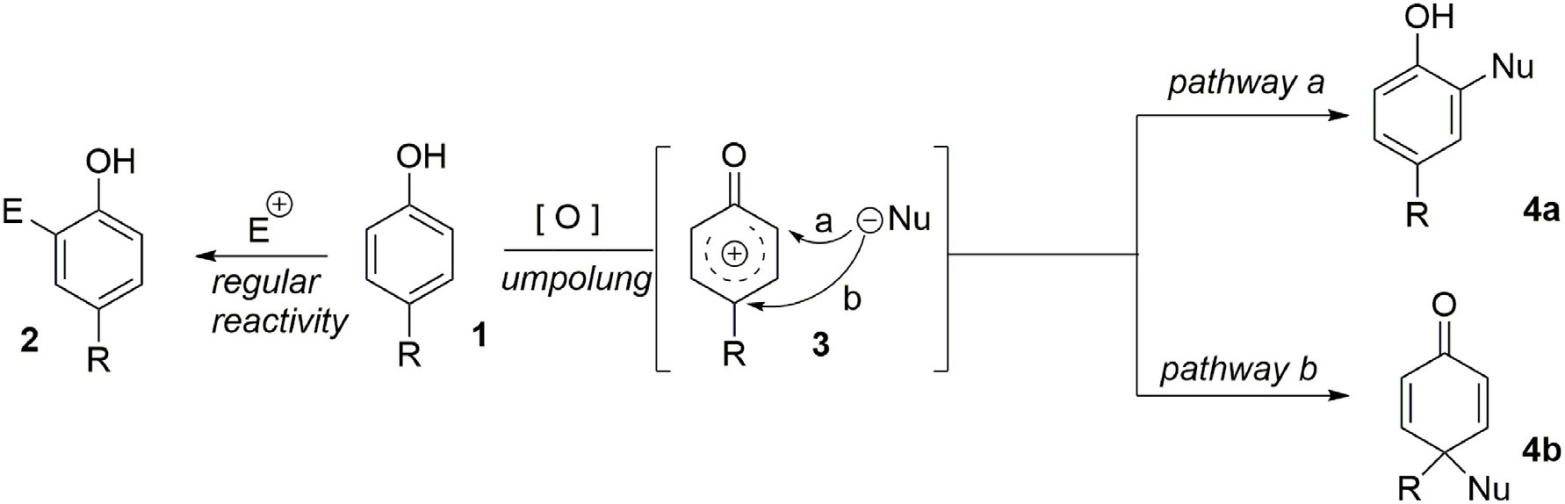
**Aromatic ring umpolung concept**.

The central theme of these syntheses is what may be termed “aromatic ring umpolung” (Guérard et al., 2010). While an electron-rich aromatic nucleus normally reacts as a nucleophile in an electrophilic pathway, suitable oxidative activation can convert it to a reactive electrophilic intermediate **3**. We assume that the formation of **3**, often described as a phenoxonium ion, is mediated by a Single-Electron Transfer (SET). It has been demonstrated that in bimolecular processes, the electrophilic species can be intercepted at two different positions, depending on the nature of the nucleophile. When a heteronucleophile is involved, the *pathway b* is observed, resulting from the attack of the unhindered heteroatom on the site bearing the larger LUMO coefficient. However, when a more hindered carbon based nucleophile is used, the *pathway a* is preferred, due to steric interactions. In this case, a more substituted aromatic compound **4a** is obtained. Finally, an intramolecular pathway remains an efficient strategy to force the nucleophile to interact at the desired position. It should be noted that when nucleophilic attack proceeds at the *para*-position (*pathway b*), a prochiral dienone system is rapidly obtained. This system may be used as a key intermediate in the total synthesis of several natural products. Indeed, this strategy provides a one-step route from an inexpensive phenol to the polyfunctionalized dienone core **4b**. Furthermore, in the presence of a carbon-based nucleophile the formation of a quaternary carbon center is observed (Figure 1).

While several oxidizing agents may be used to initiate formation of species **3** as first demonstrated in the remarkable work of Kita and coworkers (Tamura et al., 1987; Dohi et al., 2009, 2010, 2013; Ito et al., 2013), hypervalent iodine reagents (Quideau et al., 1999; Pouységu et al., 2008; Andrez et al., 2010; Liang and Ciufolini, 2010, 2011; Traoré et al., 2010; Desjardins et al., 2011; Yoshimura et al., 2012, 2013; Farid et al., 2013; Jacquemot et al., 2013) such as (diacetoxyiodo)benzene (DIB) or bis(trifluoroacetoxy)iodobenzene (PIFA) have emerged as the reagents of choice. These and similar approaches have been in use for several years, demonstrating the importance of this strategy in the total synthesis of natural products.

## Syntheses of natural compounds bearing a quaternary carbon center

**Figure 2:**
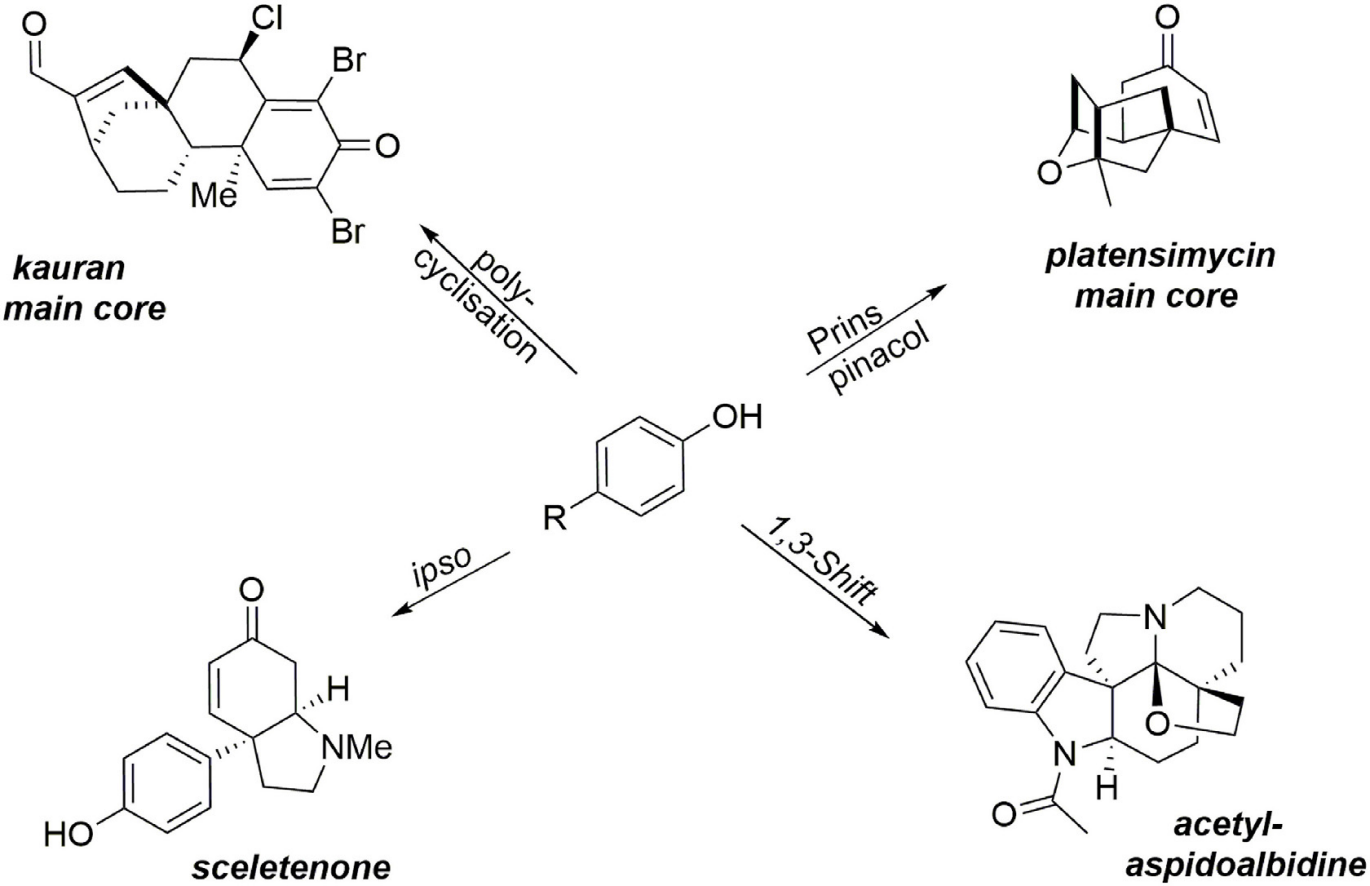
**Total syntheses of natural products bearing at least one quaternary carbon center**.

This section describes the synthesis of four compounds (two alkaloids, one heterocycle, and a diterpene) containing at least one quaternary carbon. It should be noted that the formation of such a subunit remains a challenge, particularly when stereocontrol of the quaternary center is desired. Oxidative methodologies based on phenol dearomatization mediated by an environmentally benign hypervalent iodine reagent have enabled the rapid formation of the main cores of our targets. These oxidative processes are examples of the “aromatic ring umpolung” concept, enabling the extension of several well-known aliphatic transformations involving an electrophilic intermediate to aromatic substrates. These methodologies promote the transformation of simple and inexpensive phenols into key intermediates containing a prochiral dienone system and a quaternary carbon center. For instance, a 1-3 alkyl shift process enabled synthesis of the hexacyclic alkaloid acetylaspidoalbidine, a Prins-pinacol rearrangement was a key step in formation of the main core of (−)-platensimycin, an oxidative polycyclization was used to construct the asymmetric main core of a kaurane diterpene, and the small alkaloid sceletenone was obtained through an oxidative *ipso* rearrangement (Figure 2).

### Synthesis of acetylaspidoalbidine

**Figure 3:**
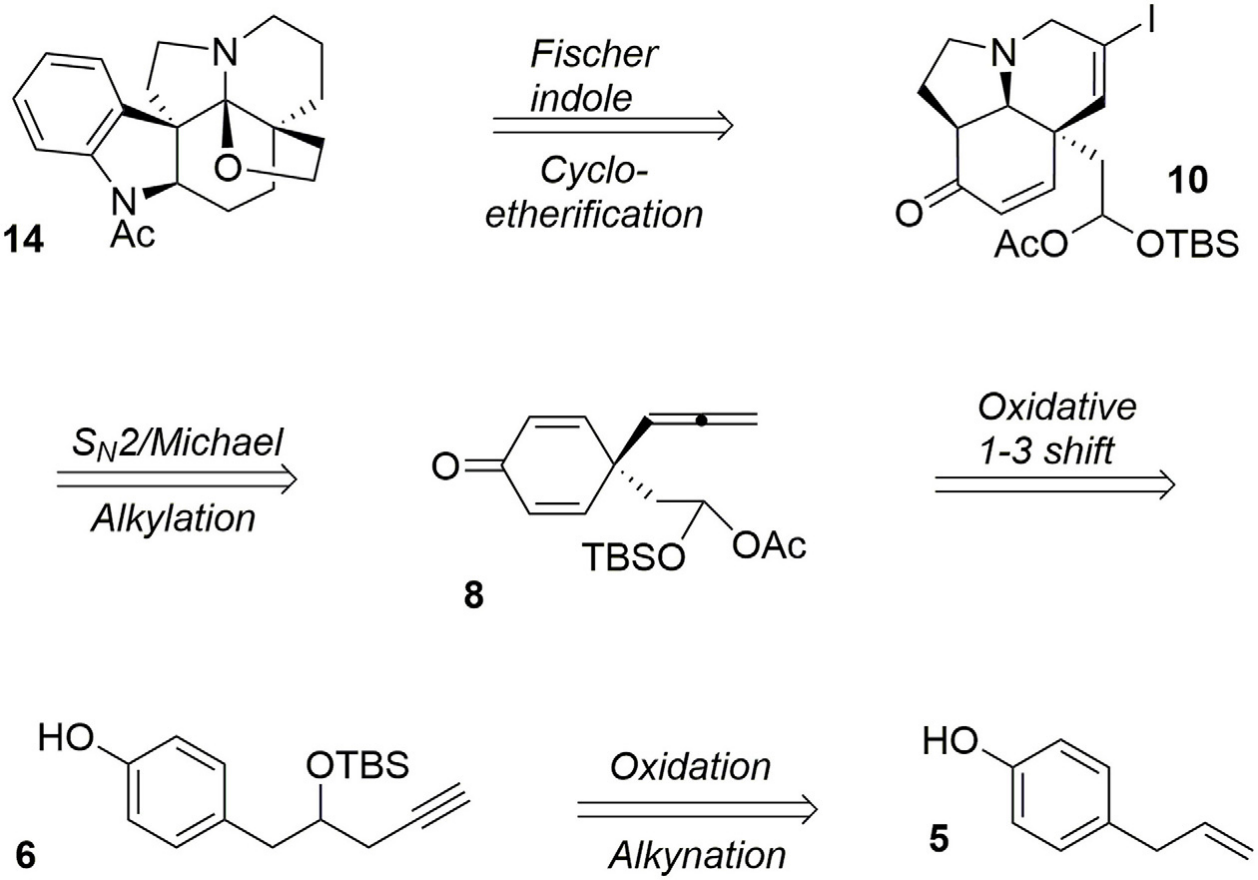
**Retrosynthesis of acetylaspidoalbidine**.

Acetylaspidoalbidine (Walser and Djerassi, 1965; Hesse, 1968; Ban et al., 1975; Honma et al., 1976; Yoshida et al., 1987; Overman et al., 1991; Campbell et al., 2010; Zhang et al., 2013) **14** is a natural alkaloid isolated from the Venezuelan tree species *aspidosperma fendleri woodson* and *aspidosperma rhombeosignatum markgraf*, and the molecule belongs to a large class of complex and bioactive alkaloids known as the aspidosperma family (Hesse, 1968). Our retrosynthesis (Guérard et al., 2012) of this molecule from the simple and inexpensive phenol derivative **5** is depicted in Figure 3.

**Scheme 1:**
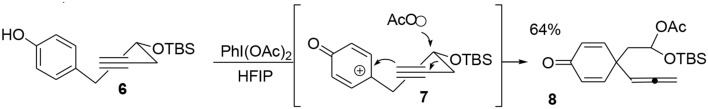
**First oxidative dearomatization process**.

One particular advantage of the umpolung activation process is its ability to adapt aliphatic transformations to aromatic systems. We have demonstrated the possibility of exploiting oxidative variations of alkyl-shift processes (Guérard et al., 2009) in particular a 1,3-propargyl migration (Guérard et al., 2012) to produce a poly-functionalized prochiral dienone **7** from a phenol derivative **6**. This approach established the first quaternary carbon center of the target as well as forming an allenyl moiety and a mixed acetal as a masked aldehyde that was used later in the synthesis (Scheme 1).

**Scheme 2:**
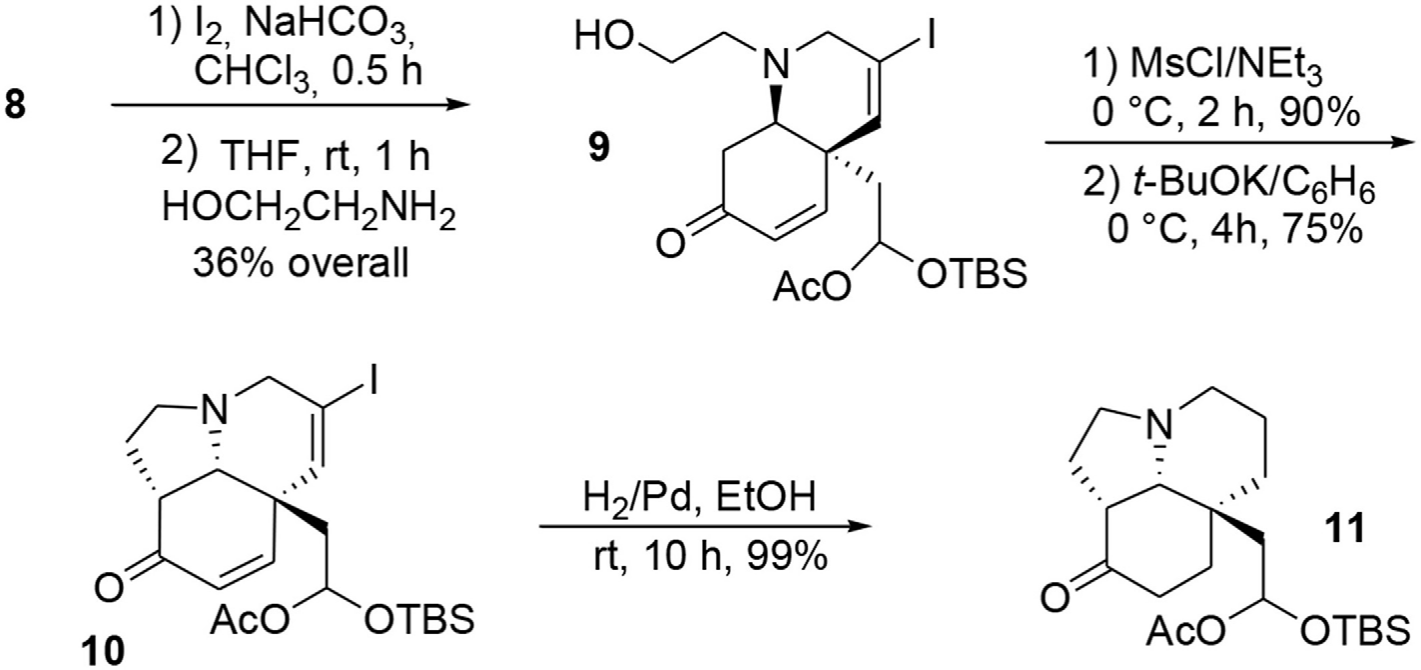
**Formation of the tricyclic core of acetylaspidoalbidine**.

Once the central dienone core was obtained, the tricyclic system of the target molecule was obtained through more typical transformations such as iodination of the allenyl moiety and subsequent treatment with ethanolamine to yield the bicycle **9** via a S_*N*_2-Michael tandem process. Further activation of the alcohol functionality in the presence of mesyl chloride followed by treatment with potassium *tert-*butoxide led to the subunit **10**. The remaining unsaturations and iodine were quantitatively removed during a final hydrogenation in the presence of palladium to produce **11**, Scheme 2.

**Scheme 3:**
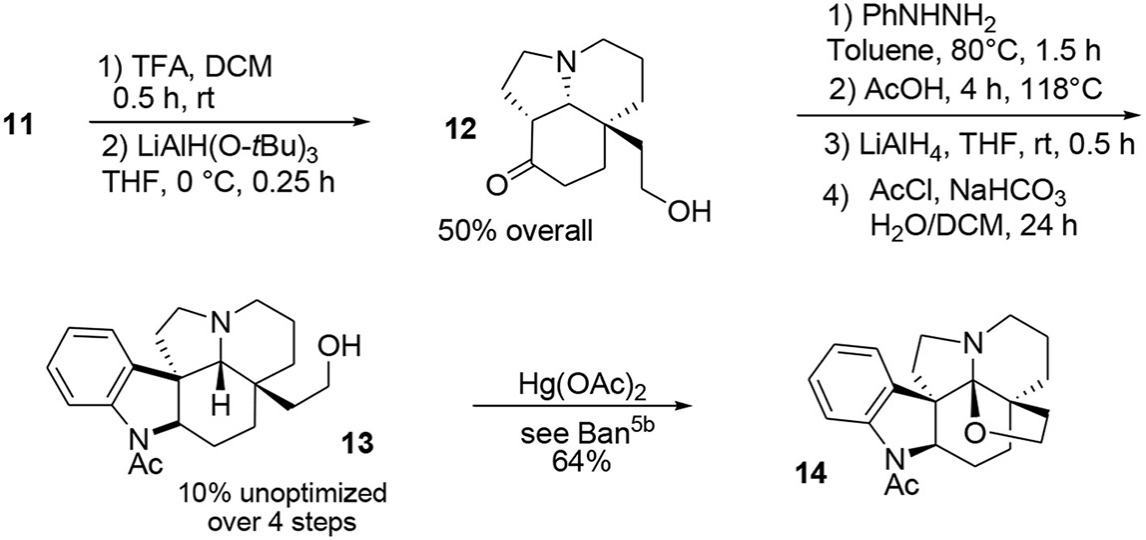
**Final steps in synthesis of acetylaspidoalbidine**.

The final portion of the synthesis was inspired by the work of Stork and Dolfini, who demonstrated that a Fischer indole-like process could be used to produce the indoline moiety of the *aspidosperma* family (Stork and Dolfini, 1963). Initial deprotection of the acetal moiety followed by selective reduction using a hindered hydride led to the primary alcohol **12**. Addition of phenylhydrazine (Fischer process) and further treatment with LiAlH_4_ afforded the pentacyclic system. Selective acetylation of the indoline nitrogen followed by oxidative cyclo-etherification (Ban et al., 1975) yielded acetylaspidoalbidine **14**, Scheme 3.

### Synthesis of (−)-platensimycin

**Figure 4:**
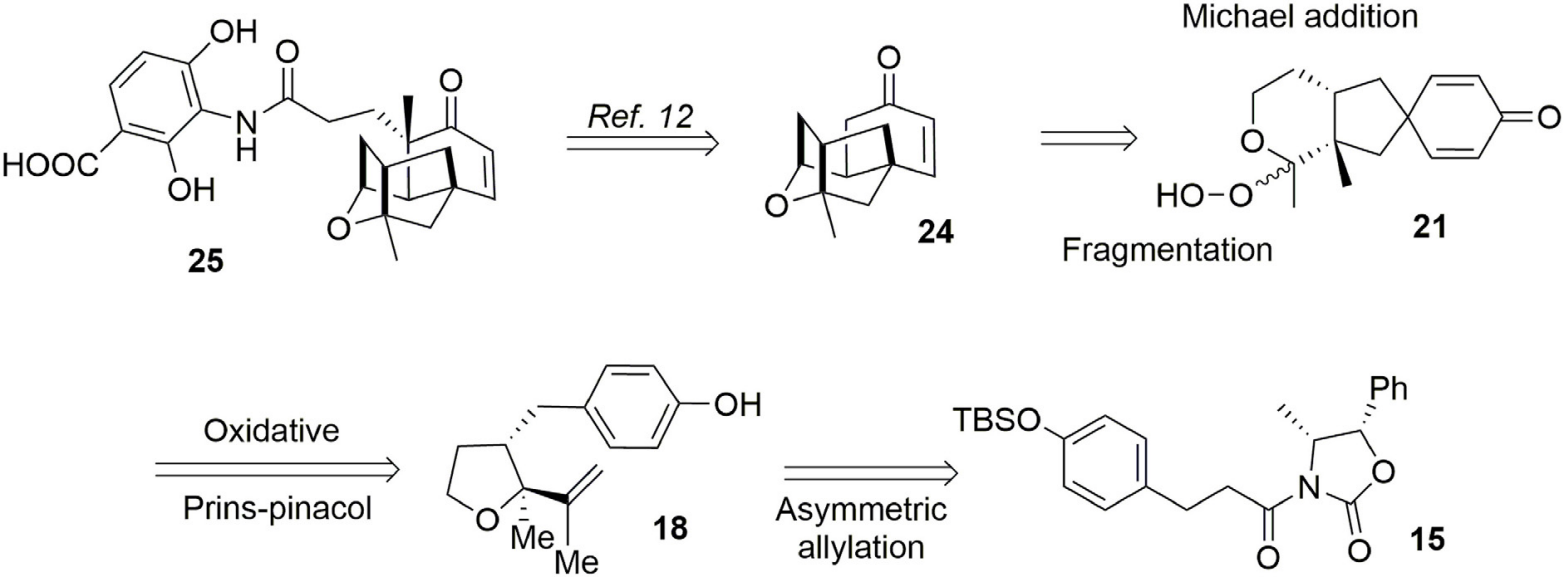
**Retrosynthesis of (−)-platensimycin main core**.

Alkyl shifts are important transformations enabling rapid and stereoselective redesign of simple architectures into polyfunctionalized scaffolds, as illustrated by the synthesis of acetylaspidoalbidine. Another important transposition resulting in formation of a complex structure is the famous Prins-pinacol process developed by Overman and coworkers (Hirst et al., 1993; MacMillan and Overman, 1995; Overman and Pennington, 2000, 2003; Lebsack et al., 2001; Lavigne et al., 2005; Overman and Velthuisen, 2006; Armstrong et al., 2007; Tang et al., 2007). Oxidative extension of this process to aromatic systems opens interesting avenues in total synthesis. In 2010, we proposed an asymmetric formal synthesis of (−)-platensimycin **25** (Beaulieu et al., 2010, 2011), a natural antibiotic isolated from a strain of *Streptomyces platensis* by Wang and Soisson in 2006 and acting as a FabF inhibitor (Wang et al., 2006). The intriguing structure and bioactivity of this compound have evoked a great deal of interest in the synthetic arena (Nicolaou et al., 2006, 2007a,b, 2008, 2009a; Lalic and Corey, 2007; Li et al., 2007; Tiefenbacher and Mulzer, 2007, 2008; Zou et al., 2007; Kim et al., 2008; Matsuo et al., 2008; Ghosh and Xi, 2009; McGrath et al., 2009; Yun et al., 2009; Eey and Lear, 2010, 2014; Palanichamy and Kaliappan, 2010; Tiefenbacher et al., 2010; Xing et al., 2010; Hirai and Nakada, 2011; Ueda et al., 2011; Zheng et al., 2011; Horii et al., 2013). As illustrated in Figure 4, we targeted structure **24**, an advanced intermediate in Nicolaou's synthesis (Nicolaou et al., 2009b) containing the main tetracyclic core of (−)-platensimycin. We envisaged producing this intermediate via a Schreiber fragmentation followed by Michael addition to dienone **21**. The latter molecule contains the desired spiranic quaternary carbon center and may be obtained from phenol **18** through an oxidative Prins-pinacol tandem process (Beaulieu et al., 2010, 2011).

**Scheme 4:**
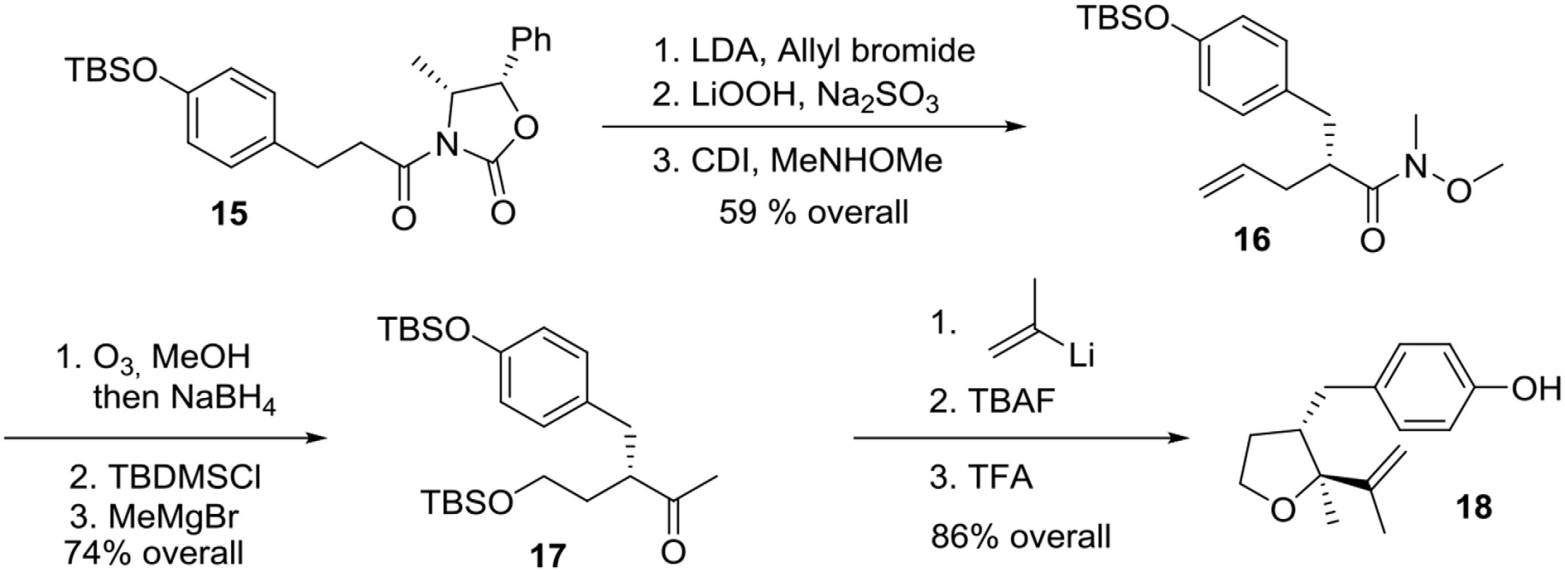
**Formation of phenol precursor**.

Preparation of the phenolic precursor **18** was achieved in nine simple steps including an Evans asymmetric allylation, an ozonolysis, and a cycloetherification under acidic conditions, Scheme 4.

**Scheme 5:**
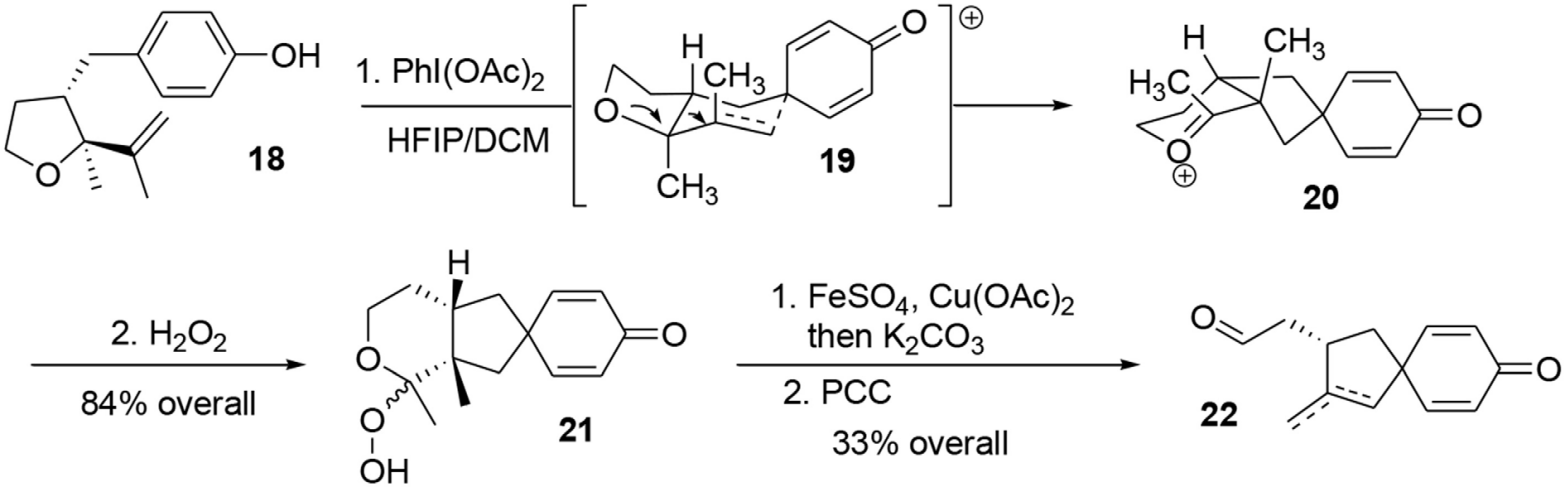
**Oxidative Prins-pinacol key step**.

Oxidation of phenol **18** using PhI(OAc)_2_ in a mixture of HFIP/DCM followed by addition of hydrogen peroxide furnished the hydroperoxyketal **21** in 64% yield as an unassigned 3:1 diastereomeric mixture (Scheme 5). During the process, the phenoxonium species underwent a stereoselective Prins-like reaction followed by a pinacol transposition through transition state **19**, leading to the intermediate **20**. This in turn reacted with hydrogen peroxide to produce the desired hydroperoxyketal **21**. It should be noted that this oxidative process generated two quaternary carbon centers in a stereoselective manner. The hydroperoxyketal was treated with Fenton reagent (Schreiber, 1980; Schreiber and Liew, 1985) (FeSO_4_) and Cu(OAc)_2_ in methanol, and subsequent addition of K_2_CO_3_ produced the primary alcohol. The alcohol was oxidized to the corresponding aldehyde using PCC. Aldehydes **22** were obtained as a 3:1 mixture of exo- and endocyclic alkenes; however, both of these compounds may be used as synthetic precursors of (−)-platensimycin, Scheme 5.

**Scheme 6:**
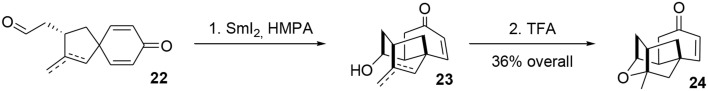
**Final steps of (−)-platensimycin main core synthesis**.

The mixture of aldehydes was subjected to Kagan's reagent (Molander, 1992; Nicolaou et al., 2009c) for stereoselective cyclization, yielding the alcohols **23**. Final treatment with trifluoroacetic acid resulted in cycloetherification to complete the formal asymmetric synthesis of (−)-platensimycin, Scheme 6.

### Stereoselective synthesis of kaurane main core

**Figure 5:**
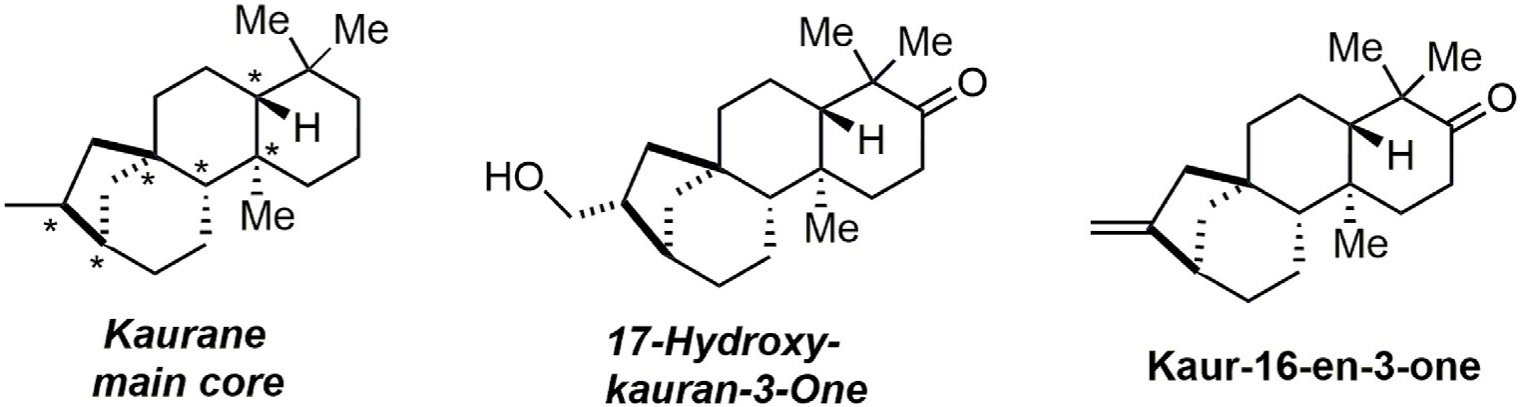
**Kauran diterpen members**.

In 2014, we reported an unprecedented tandem oxidative polycyclization-pinacol process (Desjardins et al., 2014) as a means of rapidly accessing the main core of kaurane diterpenes. These compounds belong to a large family of bioactive natural products including the examples in Figure 5. They are used in traditional Chinese medicines and exhibit bioactivities such as immunosuppression, induction of apoptosis, reduction of platelet aggregation, and anti-spasmodic effects.

**Scheme 7:**
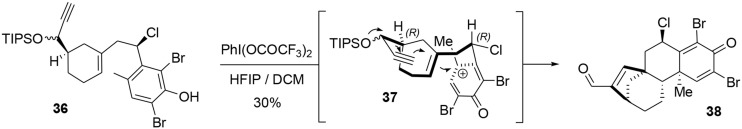
**Oxidative polycyclization-pinacol process**.

The key transformation (Scheme 7) consisted of converting the phenol **36** into the elaborated tetracyclic compound **38** in a stereoselective manner. The proposed mechanism for this reaction involved formation of the electrophilic species **37**, triggering a polycyclization cascade that concluded with a pinacol transposition and loss of the TIPS group to produce the aldehyde. The presence of the benzylic chlorine atom appears to be crucial for controlling the stereoselectivity of the polycyclization. Equatorial placement of the chlorine atom forces the side chain to react on the top face as represented in Scheme 7. *Ortho* protection of phenol **36** with bromine atoms prevented carbocation formation at these positions during oxidative activation, thus directing nucleophilic attack to the former *para* position. In this way, we were able to produce a compact and elaborated tetracyclic structure containing two quaternary carbon centers and five asymmetric carbons with total control of selectivity in one step and in 30% yield.

**Figure 6:**
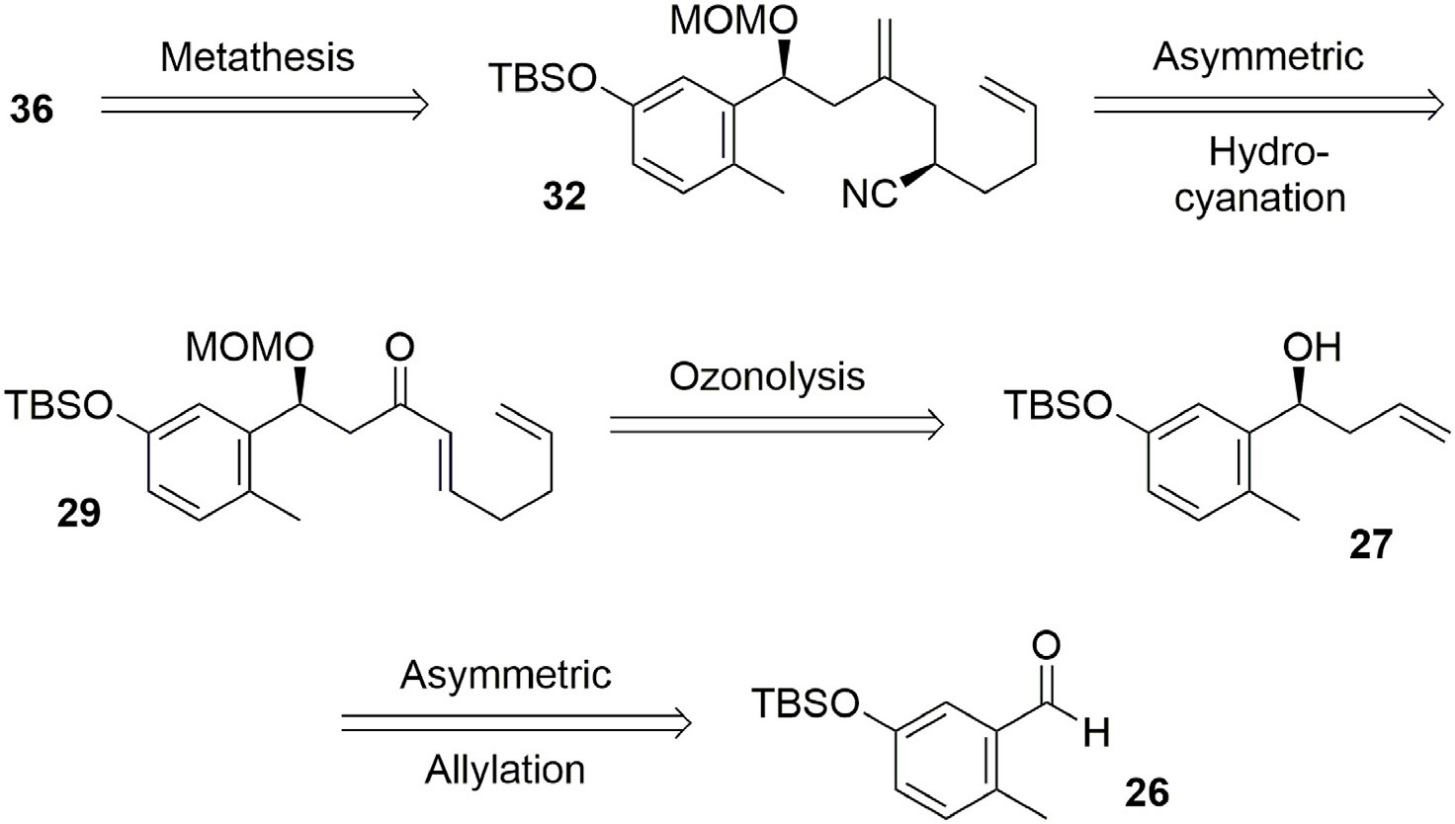
**Kaurane main core retrosynthesis**.

The envisaged retrosynthetic approach to phenol **36** is presented in Figure 6. We expected that compound **36** could be produced through ring closure metathesis of diene **32**. An asymmetric hydrocyanation would permit introduction of our second asymmetric center from Michael acceptor **29**. This enone could be easily obtained from benzylic alcohol **27** through ozonolysis followed by simple further transformations. The first asymmetric center could be generated through an asymmetric Yamamoto allylation performed on aldehyde **26**.

**Scheme 8:**
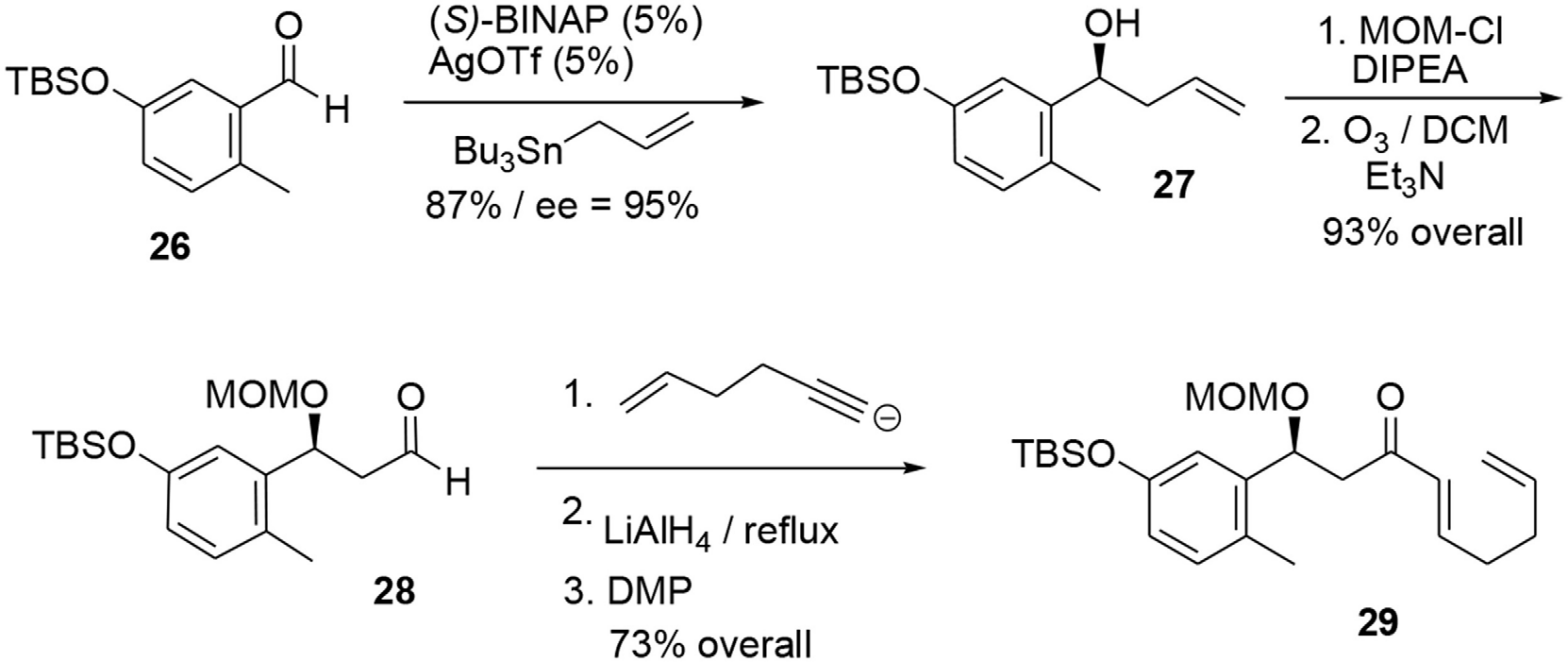
**Formation of first stereocenter**.

The synthesis of the phenolic precursor was achieved in 18 chemical steps. Initial asymmetric Yamamoto allylation of aldehyde **26** furnished the benzylic alcohol **27** in 87% yield and with good enantioselectivity (95% ee), allowing us to introduce our first asymmetric center (Scheme 8). Aldehyde **28** was obtained through ozonolysis after protection of the benzylic hydroxyl group with MOM. Michael acceptor **29** was quickly obtained through nucleophilic attack at the aldehyde followed by stereoselective reduction of the triple bond using LiAlH_4_ and oxidation of the resulting allylic alcohol using DMP.

**Scheme 9:**
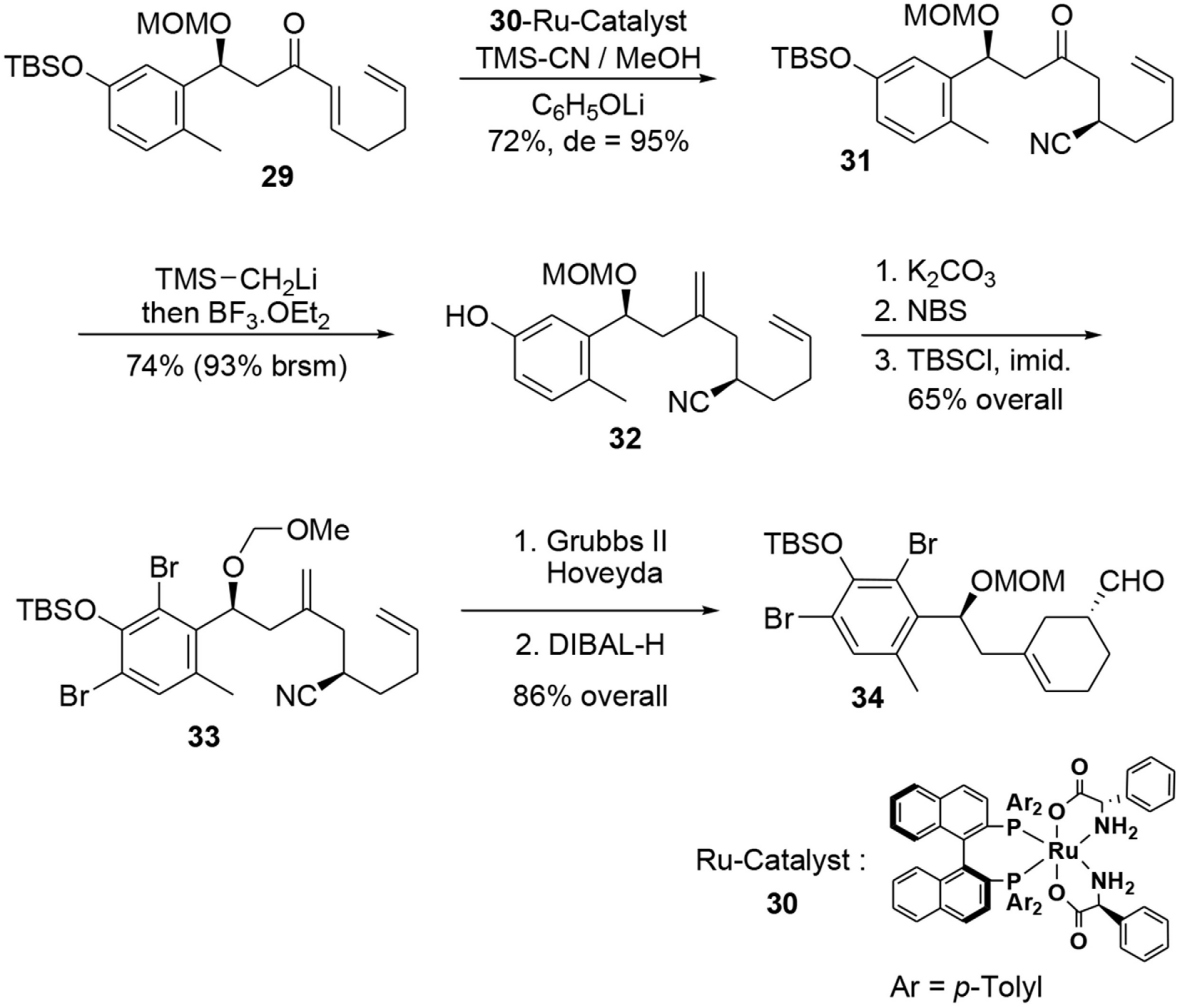
**Formation of second stereocenter**.

A further asymmetric hydrocyanation using Ohkuma's ruthenium-based catalyst (Kurono et al., 2011) **30** followed by a Peterson reaction under acidic conditions led to the corresponding diene **32** (Scheme 9). At this stage, the bromine atoms were introduced through classic electrophilic aromatic substitution and the cyclohexene moiety was generated during a ring closure metathesis reaction in the presence of a catalytic amount of Grubbs Hoveyda II. Subsequent treatment with DIBAL-H afforded the enantiopure aldehyde **34**.

**Scheme 10:**
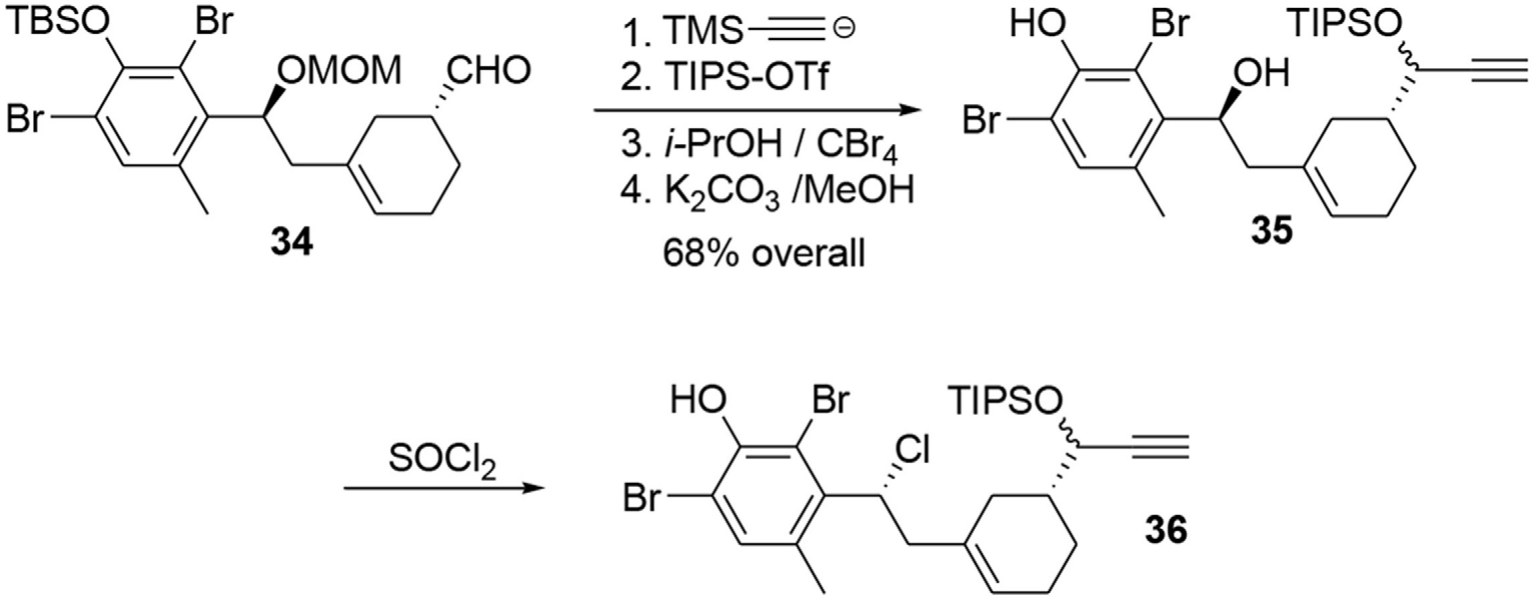
**Elaboration of phenol precursor**.

The aldehyde was trapped by addition of the lithiated anion of trimethylsilylacetylene to produce a propargylic alcohol, which was protected with a TIPS group to avoid side reactions during the polycyclization (Scheme 10). After removal of the other protecting groups, the chlorine atom was introduced by treatment of benzylic alcohol **35** with thionyl chloride.

### Synthesis of sceletenone

**Figure 7:**
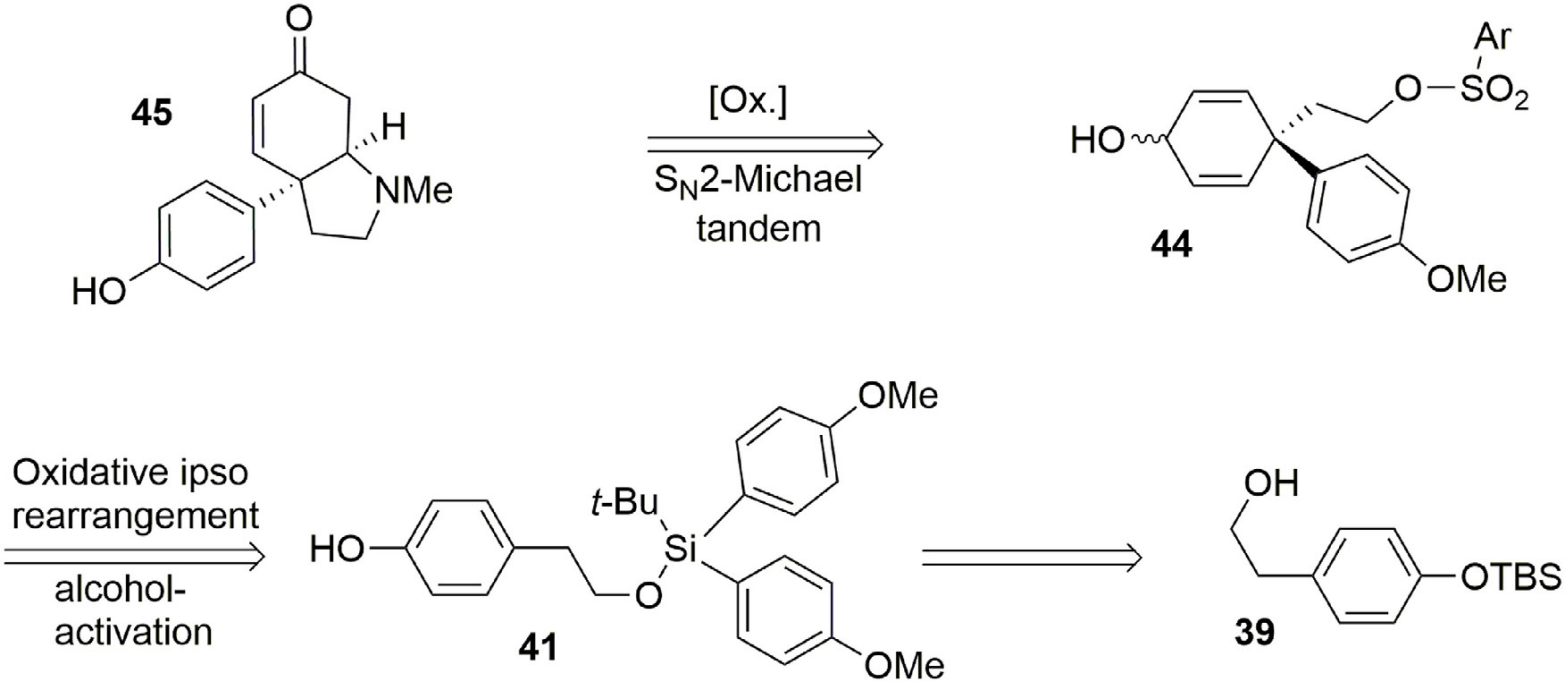
**Retrosynthesis of sceletenone**.

While transposition and polycyclization processes are eye-catching ways of producing a quaternary carbon center due to the spectacular rearrangements observed, a more direct way would involve a simple nucleophilic addition. However, in a bimolecular mode and in the presence of a hindered carbon-based nucleophile, attack on the phenoxonium ion **3** is favored at the *ortho* position (Guérard et al., 2010) (Figure 1, **pathway a**). In order to force the addition to occur at the desired *para* position, the phenol lateral chain may be used as a tether in a six-membered ring transition state mediated by an *ipso*-type rearrangement (Jacquemot and Canesi, 2012). As an illustration of this possibility, we synthesized sceletenone **45** (Jeffs et al., 1974, 1978; Sanchez et al., 1983), a small alkaloid belonging to the *amaryllidaceae* family. This molecule is isolated from the phenolic alkaloid fraction of *Sceletium strictum* and is a potent serotonin reuptake inhibitor. The natural product is isolated in racemic form, probably due to a potential aza-Michael/retro-Michael equilibrium. A retrosynthesis of this molecule is described in Figure 7.

**Scheme 11:**
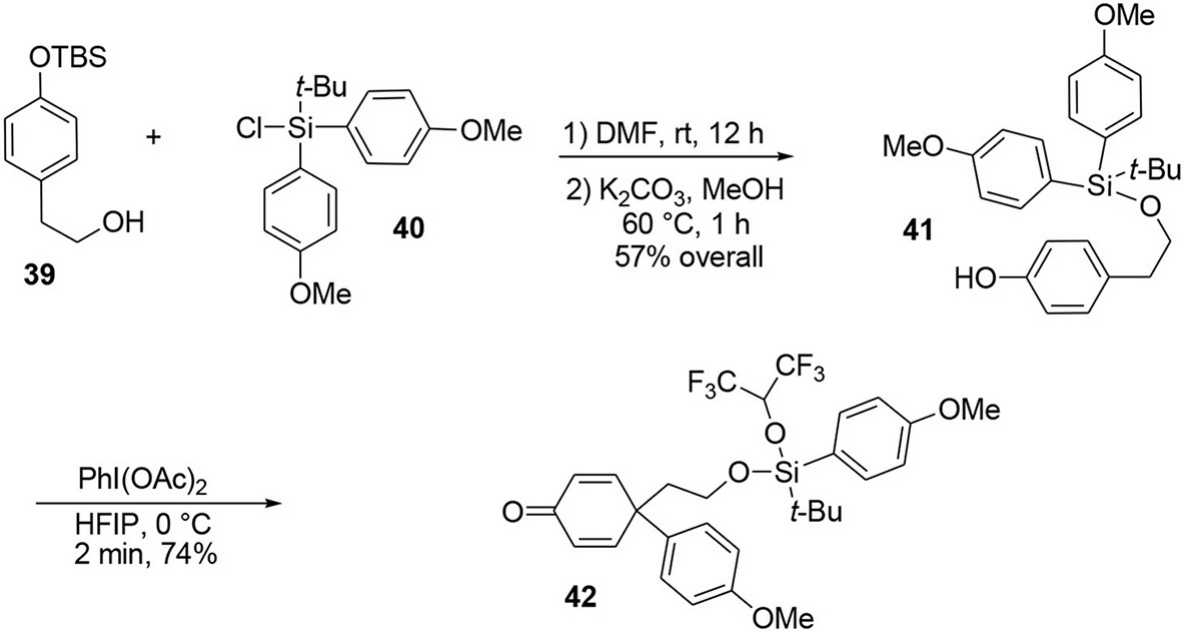
**Quaternary carbon center formation by oxidative ipso rearrangement**.

Our synthesis began with the known alcohol **39** (Nakajima et al., 2005), in which the hydroxyl group was used as a tether to introduce a carbon-based nucleophile on the lateral chain to force an intramolecular *para* attack, enabling formation of the quaternary carbon center required in the target **45**. The modified silyl group was not only used as a protecting group during the synthesis but also acted as a linker to enable the formation of an important part of the target. The modified *tert-*butyldiarylsilyl chloride **40** bearing anisole groups was prepared by addition of two equivalents of aryl-organolithium reagent to commercially available *tert*-butyltrichlorosilane. Once the silyl reagent was obtained, an oxidative *ipso*-rearrangement was performed to produce the dienone **42** containing a quaternary carbon center connected to three *sp*^2^ centers in 74% yield. Interestingly, an asymmetric silicon center was obtained by substitution of one aryl group with hexafluoropropanol, which was used as a solvent, Scheme 11.

**Scheme 12:**
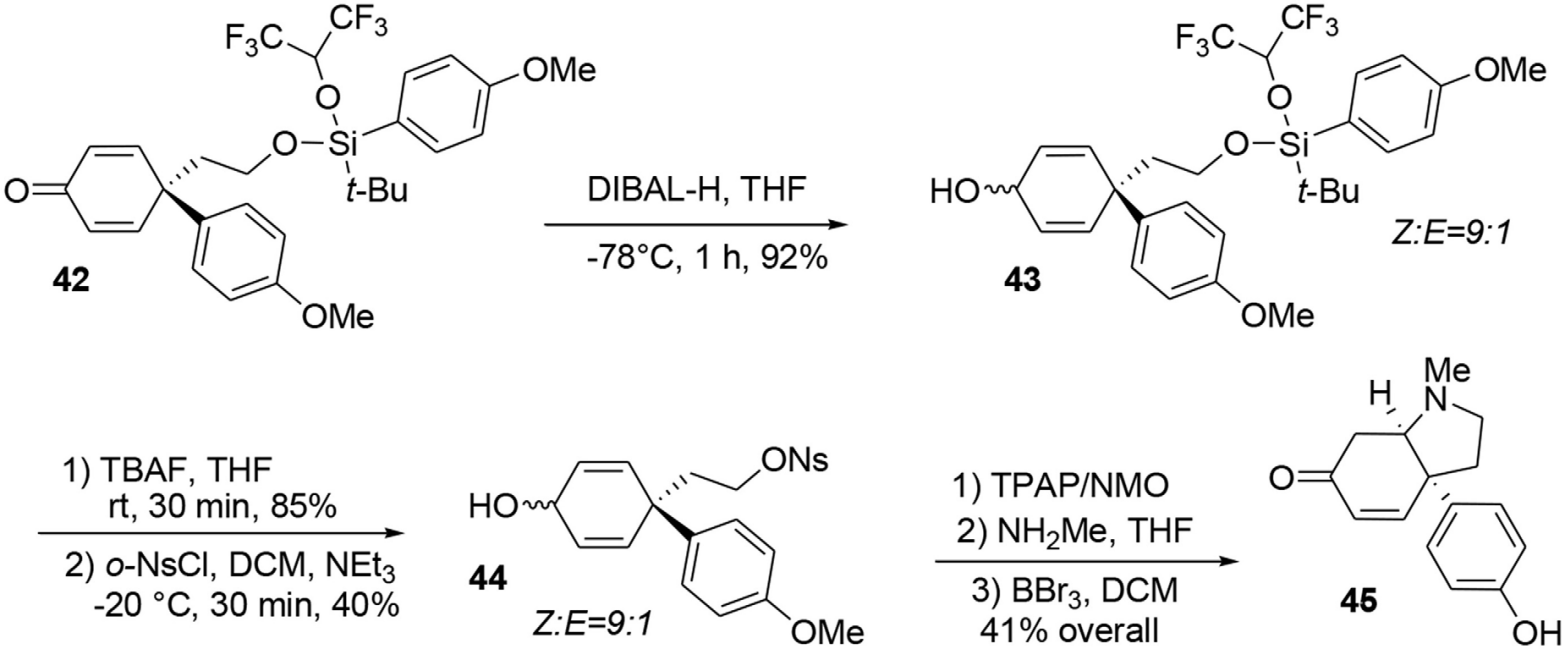
**Final steps of sceletenone synthesis**.

At this stage the formation of the pyrrolidine moiety was undertaken. A 1,2-reduction of the dienone mediated by DIBAL-H led stereoselectively to alcohol **43**. Subsequent deprotection of the silyl ether using TBAF produced the free alcohol in 85% yield. Selective activation of the primary alcohol promoted by *ortho*-nosyl chloride furnished **44** in 40% yield. Further oxidation with TPAP/NMO led to a dienone core which was treated with methylamine to afford the pyrrolidine core of sceletenone through a S_*N*_2-aza-Michael tandem process in 76% yield. The synthesis of sceletenone **45** was concluded by a final treatment with BBr_3_, Scheme 12.

## Syntheses ok alkaloids bearing a cyclic trisubstituted alkene

**Figure 8:**
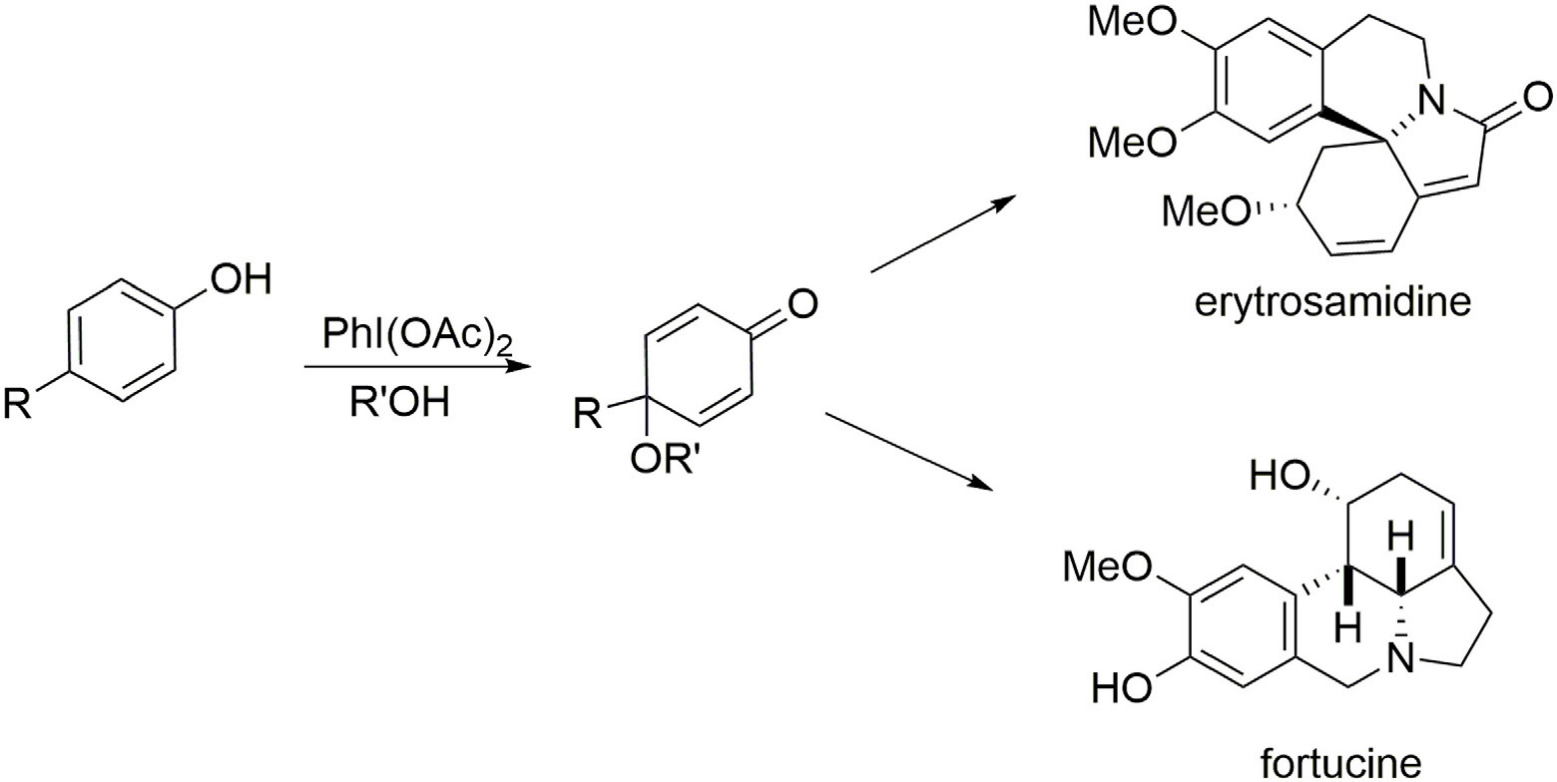
**Alkaloids bearing a cyclic trisubstituted alkene**.

One characteristic of our methodologies employing the phenol dearomatization process is the production of a carbon center at the *para* position of the original phenol that is not connected to any hydrogen atoms, either in the form of a quaternary carbon center or a substituted olefin. The second portion of this review highlights syntheses of the alkaloids erysotramidine and (−)-fortucine. Elaboration of these molecules is mediated by a two-step oxidative elimination process in which an oxygen-based nucleophile is introduced at the *para*-position to enable the dearomatization process and block the *para* position, followed by elimination of this ether moiety to insert the required polysubstituted double bond at the former *para* position (Figure 8).

### Synthesis of erysotramidine

**Figure 9:**
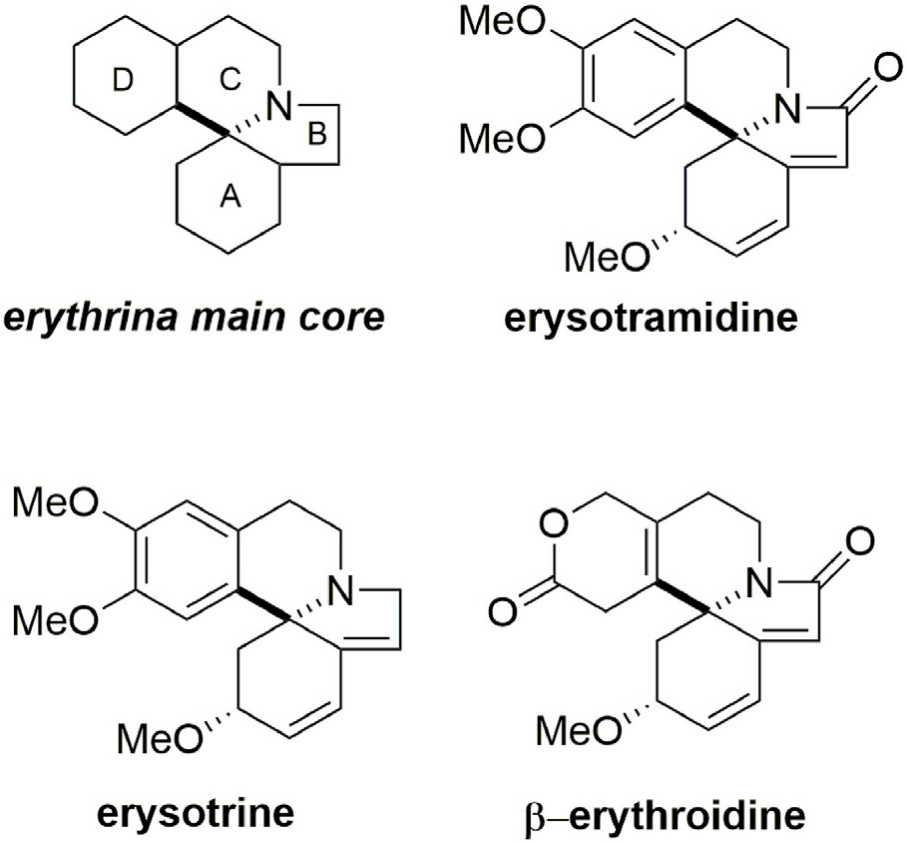
**Erythrina alkaloid members**.

Erysotramidine belongs to the erythrina alkaloid family and was isolated from tropical plants of the erythrina genus (Tsuda and Sano, 1996). These compounds contain an aza-spiran ring and exhibit several biological activities including hypotensive, sedative, anticonvulsant, and curare-like effects (Boekelheide, 1960; Hill, 1967; Dyke and Quessy, 1981; Chawla and Jackson, 1984). While some erythrina alkaloids have nonaromatic structures, others such as erysotramidine and erysotrine contain an aromatic subunit, Figure 9.

**Figure 10:**
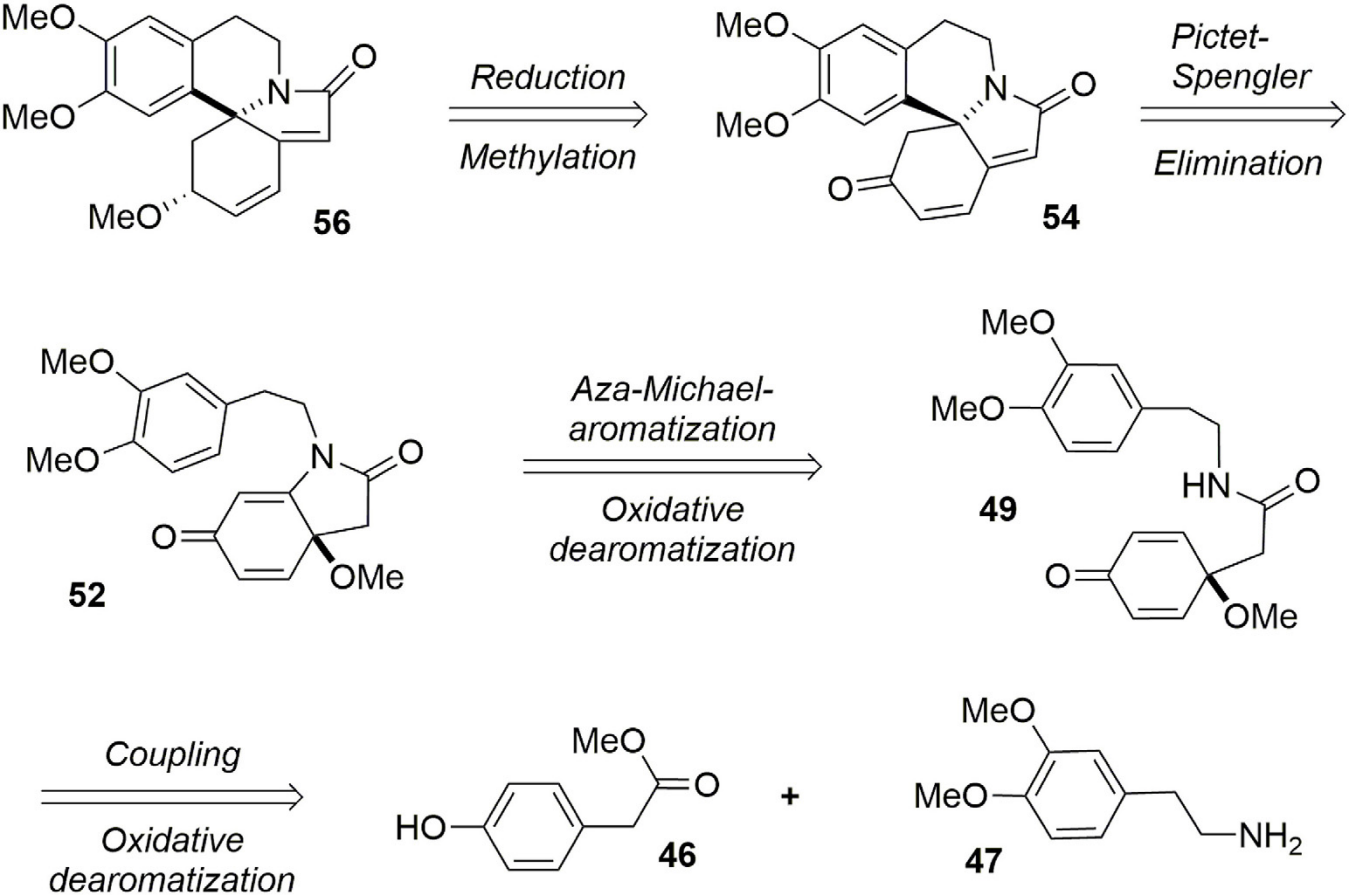
**Retrosynthesis of erysotramidine**.

Our synthesis of erysotramidine was achieved in eight steps (L'Homme et al., 2014). A retrosynthetic description is provided in Figure 10. The key steps included a stereoselective ketone reduction, a Pictet-Spengler cyclization to produce the main tetracyclic system, a novel tandem aza-Michael-rearomatization, and two oxidative dearomatization processes mediated by a hypervalent iodine reagent.

**Scheme 13:**
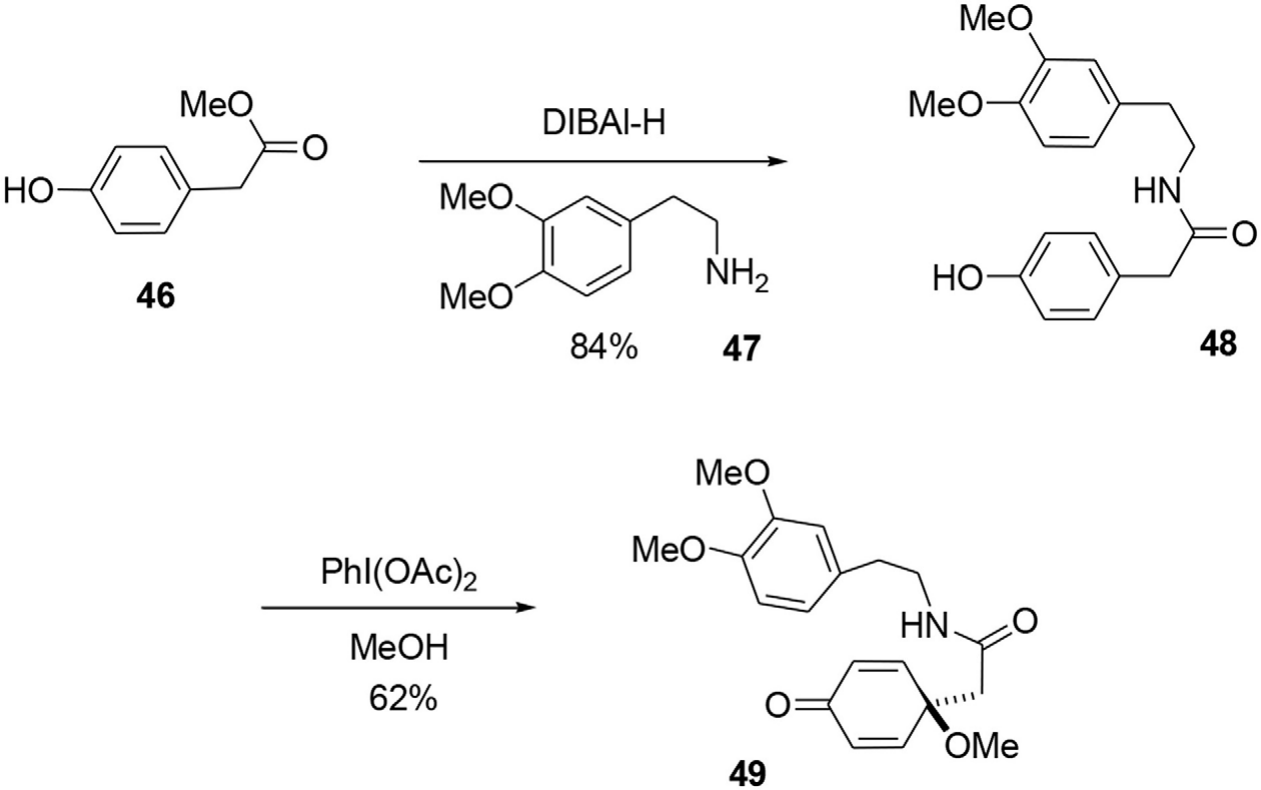
**Elaboration of the bicyclic precursor**.

Our synthesis began with a coupling reaction between phenol **46** and amine **47** in the presence of DIBAL-H to produce the corresponding amide **48**. An oxidative dearomatization using PhI(OAc)_2_ in methanol was then undertaken to transform phenol **48** into prochiral dienone **49** in 62% yield (Scheme 13).

**Scheme 14:**
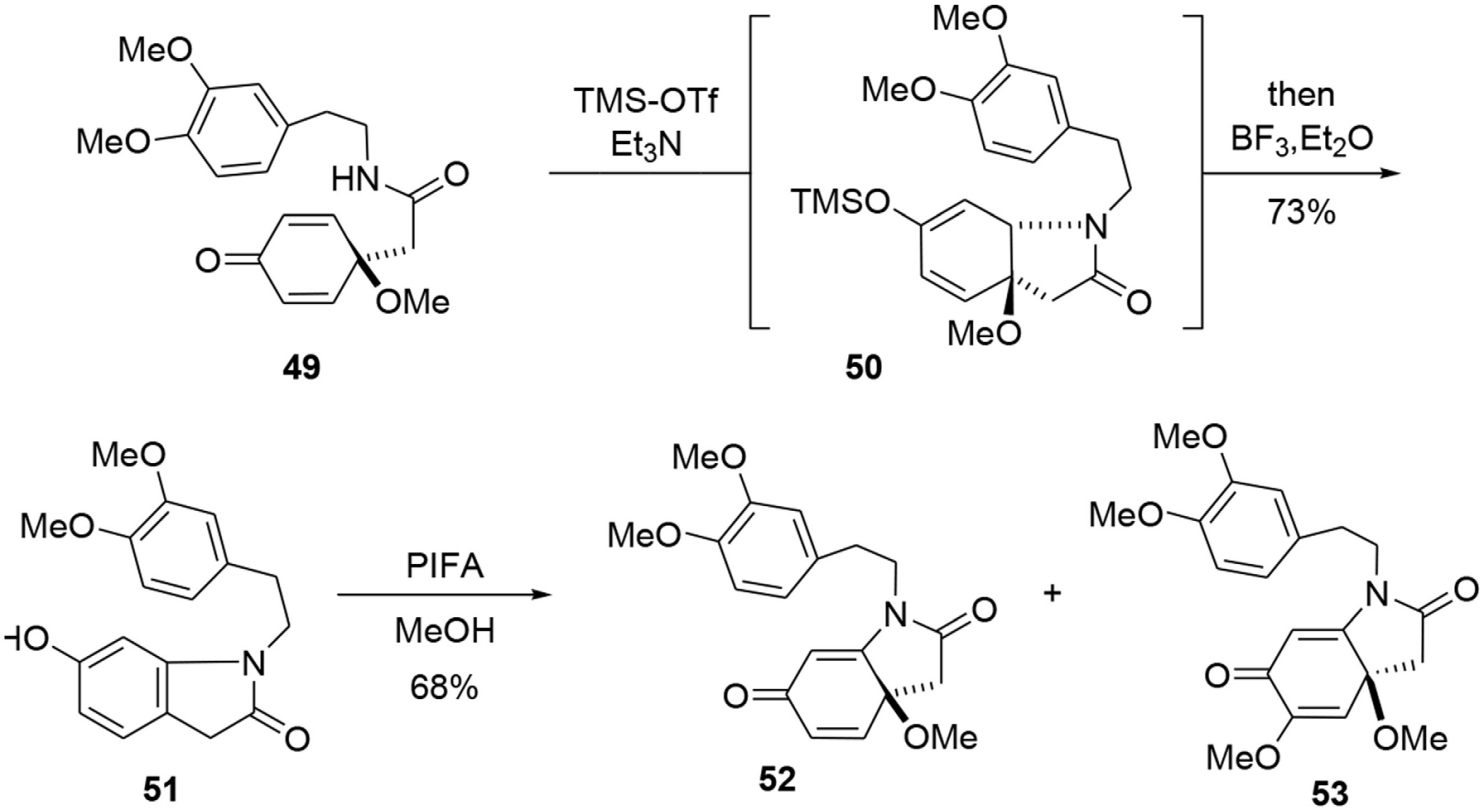
**Formation of main bicyclic system of erysotramidine**.

The bicycle **51** was subsequently obtained through a new tandem aza-Michael rearomatization process. Dienone **49** was treated with TMS-OTf in the presence of triethylamine to generate intermediate **50**. We expect the reaction first occurs through formation of an imino-ether, followed by activation of the enone moiety by the siliconium Lewis acid enabling an aza-Michael addition to yield the enol ether **50**. The methoxy group was removed by addition of BF_3_·Et_2_O in the same pot, leading to a rearomatization furnishing **51**. The phenol **51** was re-oxidized with PIFA in methanol to generate another dienone **52**. However, we observed the formation of a byproduct **53** containing two methoxy groups; this by-product was probably formed through a double activation mediated by the hypervalent iodine reagent. The desired dienone **52** was obtained in 53% yield, Scheme 14.

**Scheme 15:**
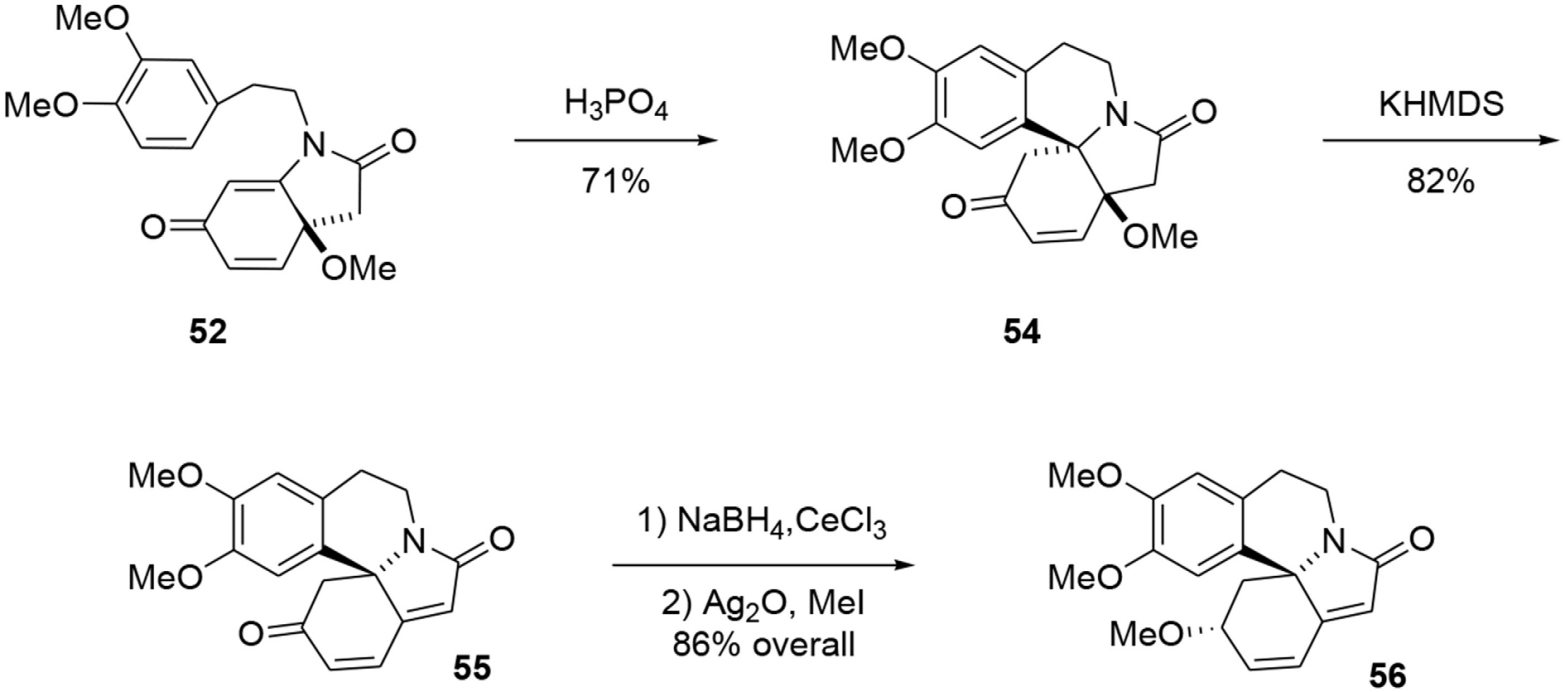
**Final steps of erysotramidine synthesis**.

The tetracyclic structure **54** was produced in a Pictet-Spengler type reaction. Under acidic conditions the aromatic moiety attacked the Michael acceptor, producing the aza-spiran in 71% yield (Maryanoff et al., 2004). At this stage, the tetracycle was treated with KHMDS to eliminate the methoxy group through an E1cB mechanism to form the polyconjugated product **55** in 73% yield, Scheme 15. The ketone functionality was reduced under Luche conditions to yield the desired alcohol with good stereoselectivity (9:1). Erysotramidine **56** was quantitatively obtained by methylation of the alcohol using Ag_2_O and iodomethane.

### Stereoselective synthesis of fortucine

**Figure 11:**
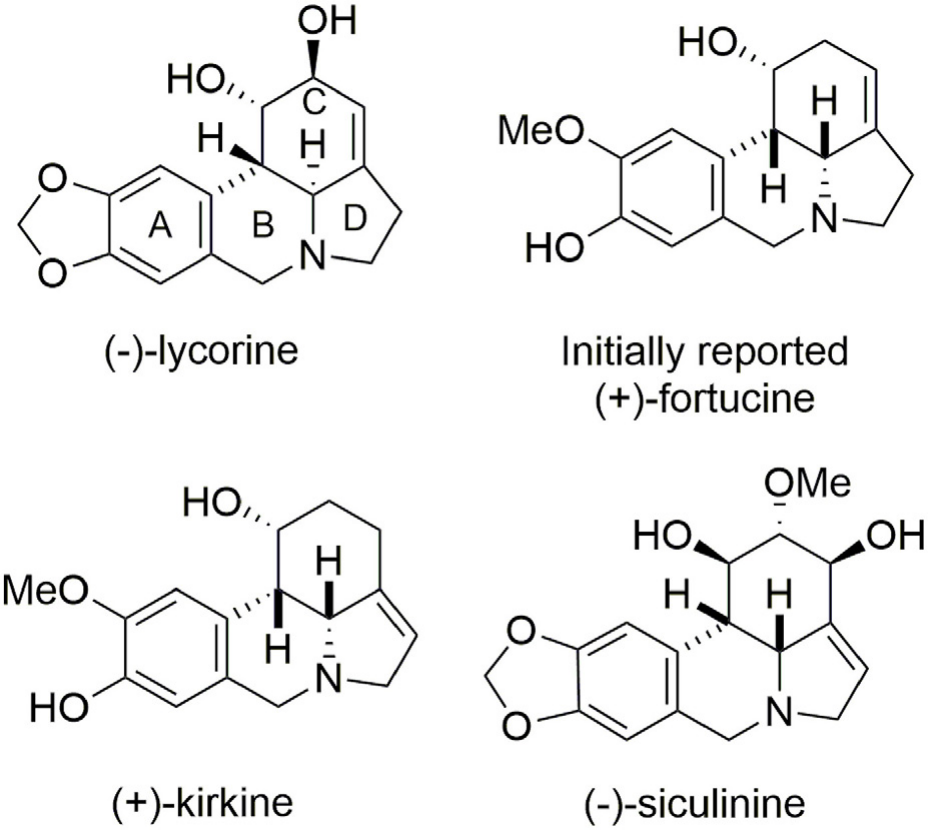
**Lycorine alkaloids**.

Fortucine belongs to the lycorine alkaloid family (Dalton, 1979) and was isolated from a fortune variety of narcissus. Lycorine alkaloids have demonstrated antitumor and antiviral activities (De Leo et al., 1973; Chattopadhyay et al., 1984; Ghosal et al., 1985; Martin, 1987). They all contain a tetracyclic pyrrolo[d,e]phenanthridine core and most (including (−)-lycorine) (Fales et al., 1955; Takagi et al., 1955; Yamada et al., 2009) present a *trans* B/C-ring junction; however, (+)-fortucine (Gorbunova et al., 1984; Tokhtabaeva et al., 1987), (+)-kirkine (Bastida et al., 1995), and (−)-siculinine (Richomme et al., 1989) contain a *cis* ring junction (Figure 11).

**Figure 12:**
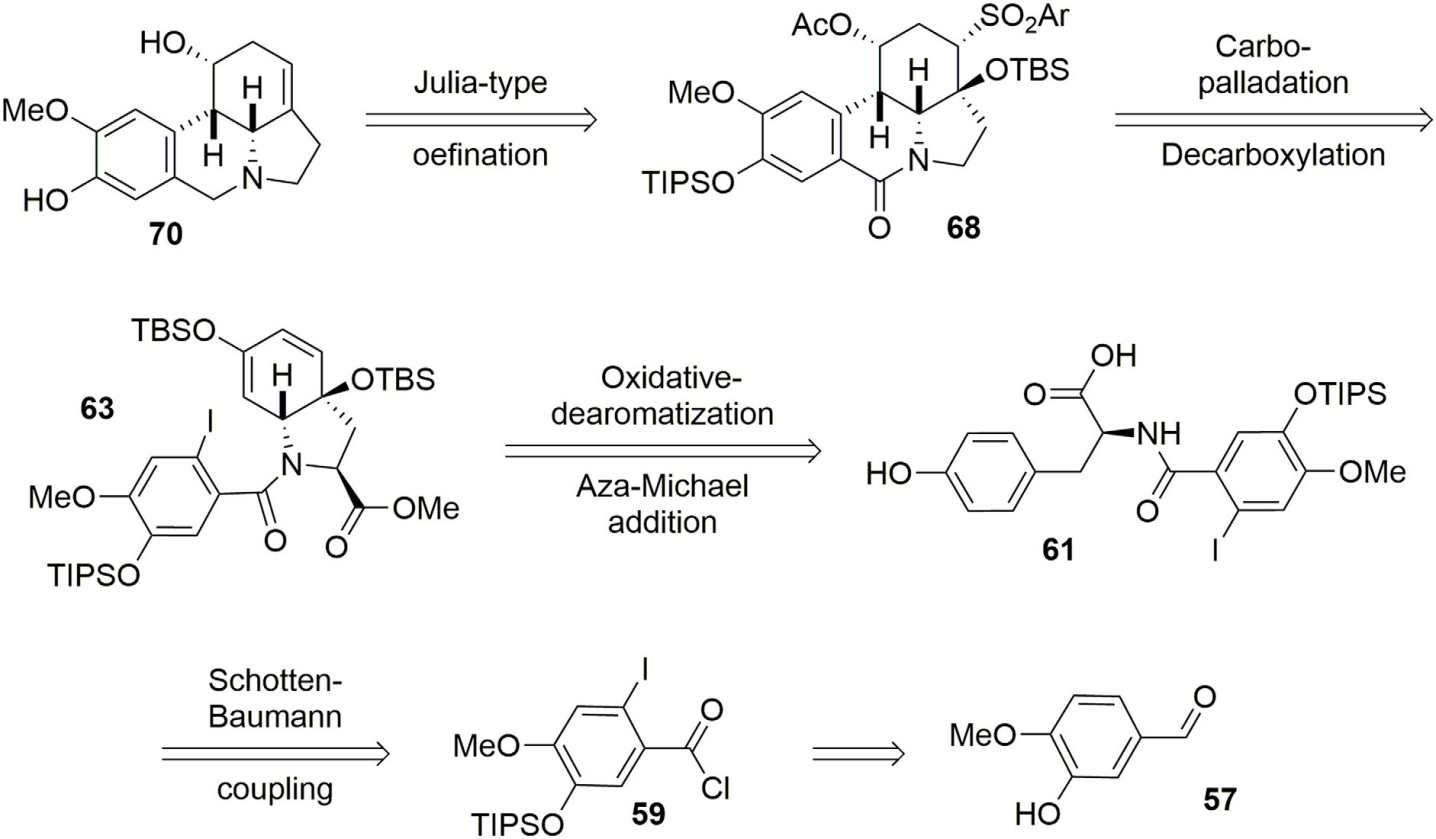
**Fortucine retrosynthesis**.

A racemic synthesis of fortucine was reported by Zard and coworkers (Biechy et al., 2008, 2009) and involved a noteworthy radical cascade reaction for formation of the main core of the target. We envisaged an enantioselective synthesis of fortucine based on the retrosynthesis presented in Figure 12. The tetracyclic structure **68** could be assembled by stereoselective carbo-palladation and decarboxylation of enol ether **63**. The latter could be obtained from a diastereoselective oxidative dearomatization – aza-Michael addition sequence applied to substrate **61** in an elegant approach first developed by Wipf and coworkers (Wipf and Kim, 1992; Wipf et al., 1995; Wipf and Li, 1999; Wipf and Mareska, 2000; Wipf and Methot, 2000). Finally, phenol **61** could be produced through a Schotten-Baumann coupling between *l*-tyrosine methyl ester and acyl chloride **59**, which is derived from the commercially available aldehyde **57**.

**Scheme 16:**
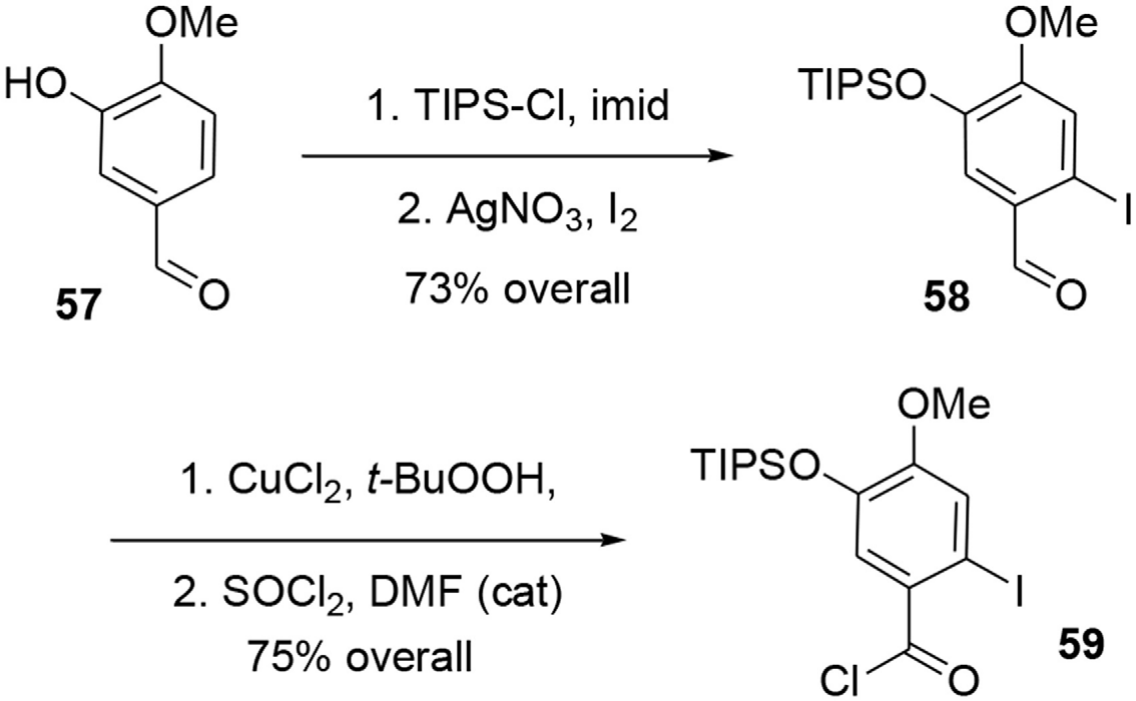
**Synthesis of aromatic moiety**.

The synthesis of intermediate **59** was achieved in four steps from commercial 3-hydroxy-4-methoxybenzaldehyde **57**. After protection of the hydroxy group using TIPSCl, the iodine atom was introduced by electrophilic aromatic substitution in the presence of I_2_ and silver nitrate. Aldehyde **58** was oxidized to the corresponding carboxylic acid (Das and Chakraborty, 2011) using *tert*-BuOOH in the presence of CuCl_2,_ and treatment with SOCl_2_ catalyzed by DMF yielded the acyl chloride **59** in 55% yield over the four steps, Scheme 16.

**Scheme 17:**
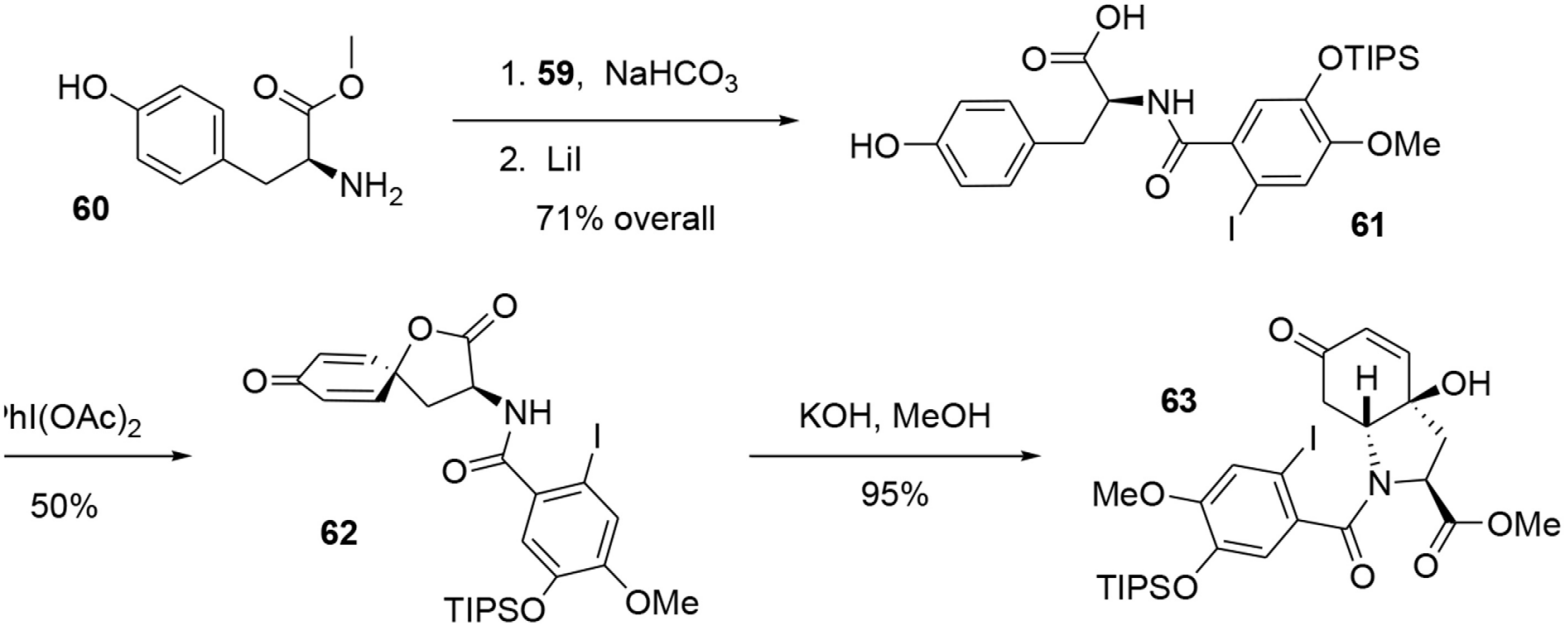
**Formation of heterocyclic system of fortucine**.

Acyl chloride **59** was coupled with *l*-tyrosine methyl ester **60** in the presence of NaHCO_3_ to generate the corresponding amide, which was subjected to mild Krapcho-like conditions to produce the intermediate **61**. At this stage, we applied the methodology developed by Wipf and coworkers (Wipf and Kim, 1992; Wipf et al., 1995; Wipf and Li, 1999; Wipf and Mareska, 2000; Wipf and Methot, 2000). This strategy consisted of activating the phenol with PhI(OAc)_2_to form the phenoxonium ion, which was trapped by the acid function to yield the spiro-lactone **62**. This was treated with KOH in methanol to cleave the lactone and promote conjugated addition of the amino group to the Michael acceptor, leading to the bicyclic enone **63**. It should be noted that the stereochemistry of that reaction was governed by the chiral center of the tyrosine (Tomioka et al., 1977), allowing us to obtain compound **63** with high stereoselectivity, Scheme 17.

**Scheme 18:**
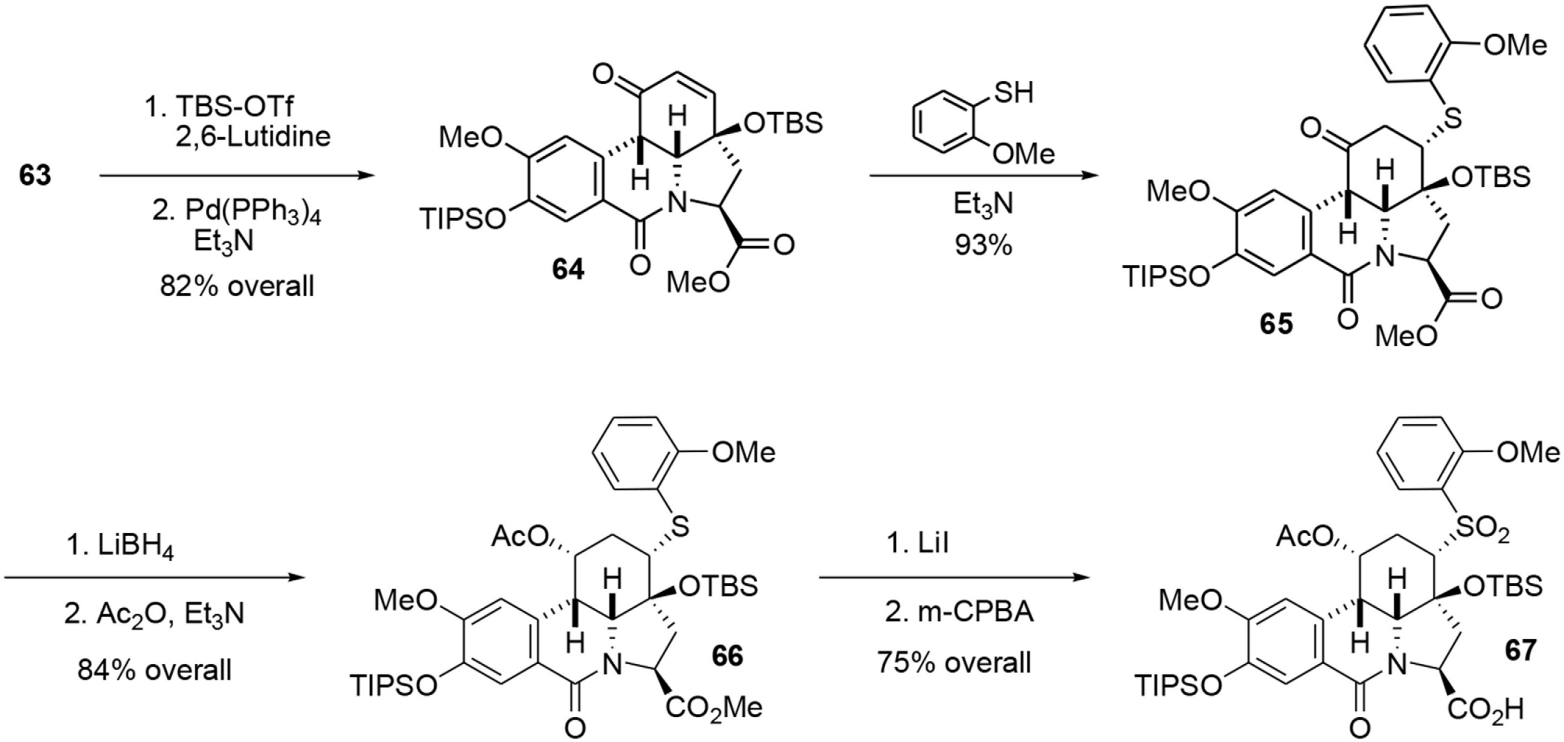
**Formation of tetracyclic portion**.

Silylation of enone **63** followed by a Heck-type carbopalladation in the presence of Pd(PPh_3_)_4_ produced the *cis* tetracyclic pyrrolo[d,e]phenanthridine **64** in 82% over two steps. We assume that the observed *cis* ring junction was a consequence of the planar geometry of the lactam moiety. It should be stressed that the tetracyclic compound **64** not only represents the main core of fortucine but also the central architecture of siculinine and kirkine. A kinetic Michael addition of 2-methoxy benzenethiol in the presence of Et_3_N formed the thioether **65**. This functionality was necessary for the planned Julia-type reaction at the end of the synthesis. An enantiopure protected alcohol was obtained by reduction of ketone **65** with LiBH_4_ followed by acetylation of the hydroxyl group under classic conditions. Methyl ester **66** was subsequently converted to a carboxylic acid under mild Krapcho-like conditions and the thioether was oxidized to the corresponding sulfone **67** by treatment with *m*-CPBA, Scheme 18.

**Scheme 19:**
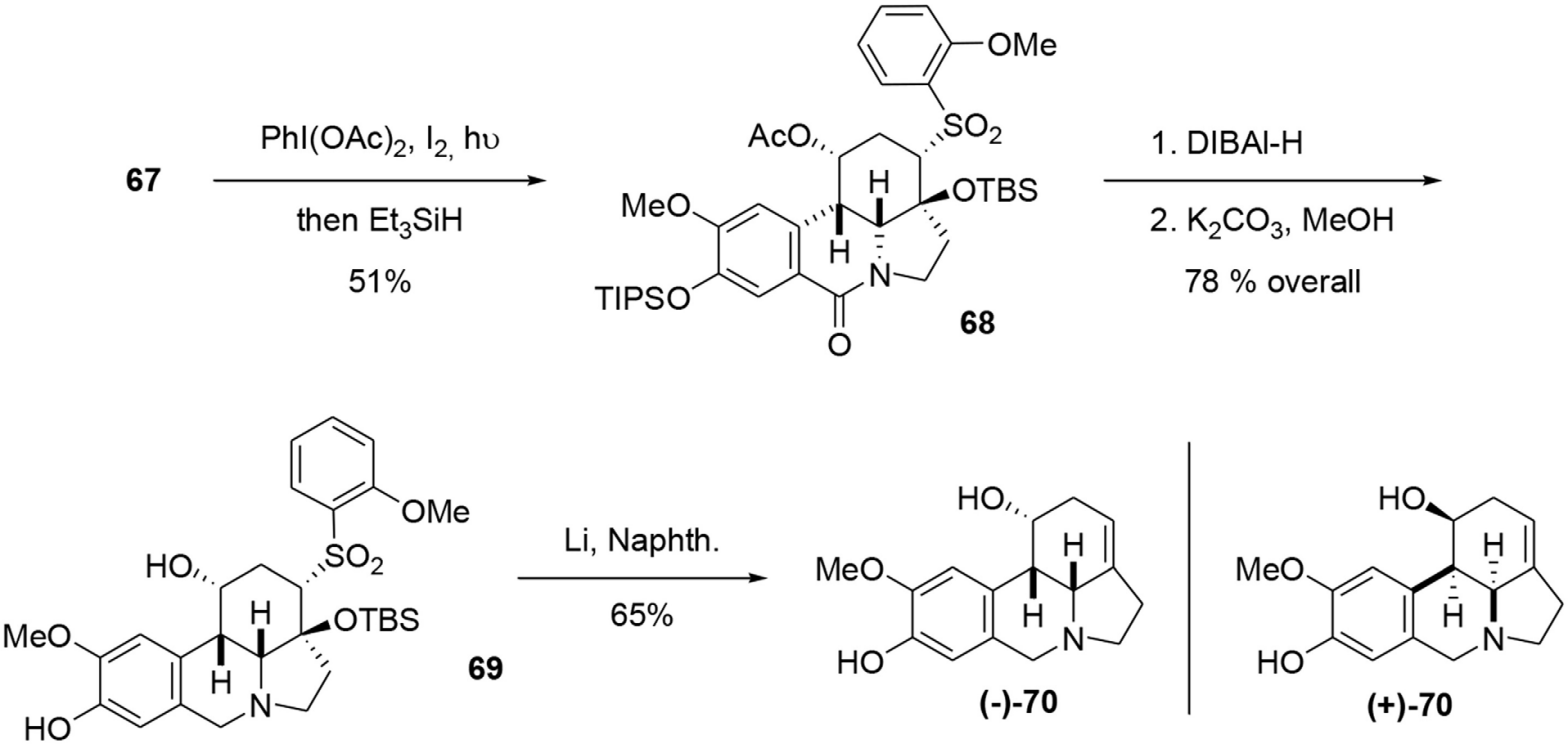
**End of synthesis and correction of absolute configuration**.

Compound **67** was decarboxylated using a photochemical process developed by Boto et al. (2000, 2001) and involving a hypervalent iodine reagent in the presence of molecular iodine. The resulting iminium ion was trapped using Et_3_SiH to yield the lactam **68**. The latter was reduced with DIBAL-H, and the TIPS and acetate groups were selectively removed. Finally, a Julia-type reaction (Julia and Paris, 1973) in the presence of Li/naphthalene resulted in formation of the desired unsaturation in 65% yield. Although the NMR and mass spectrometric data for our synthetic fortucine were in accordance with the literature (Bastida et al., 1995), we observed that the optical rotation had the opposite sign from natural fortucine. We also observed that our compound and natural fortucine had opposite Cotton effects at 285 nm. A crystallographic study of substrate **69** confirmed that we had synthesized the reported enantiomer of fortucine (Beaulieu et al., 2014), meaning that the natural product is actually the mirror image of the structure that had previously been described, Scheme 19.

## Conclusion

We have summarized several total natural product syntheses developed by our group. All of the syntheses highlight the importance of hypervalent iodine reagents as well as the utility of the “aromatic ring umpolung” concept. Indeed, one to two key steps in each synthesis involved hypervalent iodine reagents. A main challenge that requires to be addressed in a near future is the development of an efficient stereoselective methodology enabling to desymmetrize prochiral systems into enantiopur precursors for asymmetric synthesis. Hypervalent iodine chemistry is in constant growth, and these strategies are only examples of the expanding literature on the applications of hypervalent iodine reagents in the synthesis of natural products.

### Conflict of interest statement

The authors declare that the research was conducted in the absence of any commercial or financial relationships that could be construed as a potential conflict of interest.
